# Metal Ion Binding to the Amyloid β Monomer Studied by Native Top-Down FTICR Mass Spectrometry

**DOI:** 10.1007/s13361-019-02283-7

**Published:** 2019-07-26

**Authors:** Frederik Lermyte, James Everett, Yuko P. Y. Lam, Christopher A. Wootton, Jake Brooks, Mark P. Barrow, Neil D. Telling, Peter J. Sadler, Peter B. O’Connor, Joanna F. Collingwood

**Affiliations:** 1 School of Engineering, University of Warwick, Coventry, CV4 7AL UK; 2 Department of Chemistry, University of Warwick, Coventry, CV4 7AL UK; 3 Institute for Science and Technology in Medicine, Keele University, Stoke-on-Trent, Staffordshire ST4 7QB UK

**Keywords:** Native mass spectrometry, Amyloid beta, Peptide-metal complex, Native top-down, Mass spectrometry, Electron capture dissociation, Collision-induced dissociation, Infrared multiphoton dissociation, Radical-directed dissociation

## Abstract

Native top-down mass spectrometry is a fast, robust biophysical technique that can provide molecular-scale information on the interaction between proteins or peptides and ligands, including metal cations. Here we have analyzed complexes of the full-length amyloid β (1-42) monomer with a range of (patho)physiologically relevant metal cations using native Fourier transform ion cyclotron resonance mass spectrometry and three different fragmentation methods—collision-induced dissociation, electron capture dissociation, and infrared multiphoton dissociation—all yielding consistent results. Amyloid β is of particular interest as its oligomerization and aggregation are major events in the etiology of Alzheimer’s disease, and it is known that interactions between the peptide and bioavailable metal cations have the potential to significantly damage neurons. Those metals which exhibited the strongest binding to the peptide (Cu^2+^, Co^2+^, Ni^2+^) all shared a very similar binding region containing two of the histidine residues near the N-terminus (His6, His13). Notably, Fe^3+^ bound to the peptide only when stabilized toward hydrolysis, aggregation, and precipitation by a chelating ligand, binding in the region between Ser8 and Gly25. We also identified two additional binding regions near the flexible, hydrophobic C-terminus, where other metals (Mg^2+^, Ca^2+^, Mn^2+^, Na^+^, and K^+^) bound more weakly—one centered on Leu34, and one on Gly38. Unexpectedly, collisional activation of the complex formed between the peptide and [Co^III^(NH_3_)_6_]^3+^ induced gas-phase reduction of the metal to Co^II^, allowing the peptide to fragment via radical-based dissociation pathways. This work demonstrates how native mass spectrometry can provide new insights into the interactions between amyloid β and metal cations.

**Electronic supplementary material:**

The online version of this article (10.1007/s13361-019-02283-7) contains supplementary material, which is available to authorized users.

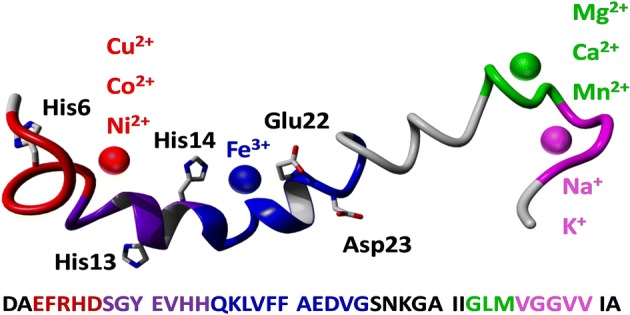

## Introduction

Gaining insight into protein-protein, protein-ligand, and protein-metal interactions at the molecular level is critical for understanding the biological function of proteins. For example, it has been estimated that metalloproteins alone make up more than a third of all proteins [1, 2]. Widely used methods for elucidating such interactions include nuclear magnetic resonance (NMR) spectroscopy and X-ray crystallography. However, these biophysical techniques typically are limited in terms of sample throughput, and NMR in particular often samples ensemble averages if multiple interaction regions/stoichiometries coexist, depending on the timescales of exchange.

Native mass spectrometry (MS) is a fast, robust method that allows study of intact noncovalent protein-protein and protein-ligand/metal complexes in the gas phase [3–5]. While the lack of a hydrophobic effect in the absence of water originally led to speculation that protein ions in vacuo would turn “inside out” [6], it is currently believed that instead they retain many aspects of their native structure, including overall shape, complex stoichiometry, and binding of ligands, even those that are hydrophobically bound [7–10]. Combined with top-down fragmentation, native MS provides a powerful tool for obtaining information about higher-order structure, including subunit connectivity and ligand binding site(s) [11–13]. Often, binding sites can be determined with single-residue specificity, although this requires extensive backbone cleavage in the region of interest, which is often not achieved in practice. This technique is also ideally and uniquely suited for systems where multiple states coexist, as a specific precursor is almost always selected based on its mass-to-charge (*m*/*z*) ratio.

In this work, we have used high-resolution Fourier transform ion cyclotron resonance (FTICR) mass spectrometry to gain insight into the sequence regions involved in peptide-metal interactions. We have used electron capture dissociation (ECD), infrared multiphoton dissociation (IRMPD), and collision-induced dissociation (CID), to determine the peptide-metal interaction sites. In this type of “native top-down” study [14], it is always a concern whether the binding site remains the same during the dissociation process. It has been shown that the increase in internal energy during CID can lead not only to loss but also to migration of a noncovalently bound ligand [15]. ECD has been used successfully to study binding sites of anticancer complexes to biomolecules [16–21]; however, the charge reduction inherent to ECD in some cases is capable of disrupting ion-dipole interactions and this mechanism can also induce ligand migration [22]. While, therefore, there are indications that results from each of our three dissociation methods in isolation should not be fully accepted a priori, consistent results from all three methods allow confident identification of the true gas-phase binding region, given the fundamentally different fragmentation mechanisms involved. An important objective of this study was to investigate to what extent results from the three methods differ. This is because consistency between all three across the relatively broad range of metals tested here would be a strong indication that each method in isolation can be used to identify metal cation binding regions, and that it would generally suffice in such a study to use the method most conveniently available and/or that would lead to the most efficient dissociation.

Amyloid β (1-42) was used as a model peptide in this work. Misfolding and accumulation of this peptide, particularly the 40- and 42-residue isoforms, are widely believed to play a major role in the pathogenesis of Alzheimer’s disease, the most prevalent neurodegenerative disease worldwide. It is less widely recognized that interactions between certain trace metals and the peptide play an important role in disease etiology [23–27]. Such interactions have been studied in both in vitro and ex vivo samples, using a range of techniques, including fluorescence assays [27–29], NMR [28–30], Raman [26], and synchrotron X-ray spectromicroscopy [31–33]. In the current work, the metal ions Na^+^, K^+^, Mg^2+^, Ca^2+^, Mn^2+^, Co^2+^, [Co(NH_3_)_6_]^3+^, Ni^2+^, Cu^2+^, and Fe^3+^ were reacted with buffered aqueous solutions of the full-length peptide. These metal cations were mainly chosen because they easily and reproducibly lead to the formation and detection of amyloid-metal complexes under the in vitro conditions used here, but with the exception of [Co(NH_3_)_6_]^3+^, they are also all physiologically or pathophysiologically relevant [24, 31, 33–41]. Although there is widespread interest in the possible involvement of Zn^2+^ and Al^3+^ in neurodegeneration on account of their known or suspected dysregulation functions in Alzheimer’s disease, we were unable to obtain consistent MS data for binding of these metal cations to amyloid β (1-42), and so no results are reported here. We believe this is due to their complicated hydrolytic chemistry at physiological pH (formation of hydroxide-bridged oligomers, etc.), as we will illustrate for Fe^3+^. Although rarely mentioned in the context of AD, Co is included and potentially of interest following a reported increase in brain Co in AD patients versus healthy controls [42]. Interest in Ca has primarily focussed on signalling, and in the context of amyloid accumulation, we highlight our recent observation of diverse calcium biomineral phases within amyloid plaques isolated from humans with AD [33].

## Experimental

MS experiments were performed on a 12 T solariX quadrupole/FTICR instrument (Bruker Daltonik GmbH, Bremen, Germany) equipped with an infinity cell and advanced dissociation methods, including electron capture dissociation and infrared multiphoton dissociation. Ions were introduced using nano-ESI in positive ion mode and externally accumulated in a hexapole collision cell before being transferred to the ICR cell. The ESI source used 1.2-mm thin-walled glass capillaries (World Precision Instruments, Hitching, UK) that were pulled in-house to obtain tips of ca. 1-μm orifice diameter with a P97 Flaming/Brown type micropipette puller (Sutter Instrument Co., Novato, CA, USA). For CID and ECD experiments, precursor ions were selected in the quadrupole of the solariX instrument. For IRMPD, in-cell isolation was performed using the correlated harmonic excitation fields (CHEF) method [43], with a notch width of 3 to 5 *m*/*z*, and excitation energy of 55%. ECD was performed by generating electrons from a heated hollow cathode, using a current of 1.5 A. IRMPD was performed using a Synrad 48-2 25 W CO_2_ laser with a 10.6-μm wavelength operated at 50% power. Data analysis was performed using Bruker Compass DataAnalysis 4.1, and peaks were assigned manually. Detailed peak assignment tables can be found in Supporting Information S7. To preserve native peptide-metal interactions, samples were prepared in Milli-Q H_2_O containing 25 mM ammonium acetate (pH 7.4). Amyloid β (1-42) (monoisotopic mass 4511.27 Da) was purchased from Bachem (Bubendorf, Switzerland). The concentration of the peptide was kept constant at 12.5 μM. NaCl, KCl, MgSO_4_, CaCl_2_, MnCl_2_, [Co(NH_3_)_6_]Cl_3_, CoCl_2_, CuSO_4_, NiCl_2_, FeCl_3_, and nitrilotriacetic acid were purchased from Sigma (Dorset, UK). Transmission electron microscopy (TEM) images were acquired using a JEOL 2010 microscope operated at 200 kV, and negative staining was performed using uranyl acetate.

## Results and Discussion

### The non-traditional buffer required for native MS of amyloid-metal complexes did not prevent normal fibril formation

As a first step, we briefly investigated the use of different buffers to study amyloid-metal interactions by mass spectrometry. Most literature reports do not employ MS and have used conventional molecular biology buffers, such as phosphate-buffered saline (PBS), and performing MS using these buffers would have the benefit of eliminating one potential source of discrepancy when comparing our results to the literature. Unfortunately, these conventional buffers contain significant levels of non-volatile cations, such as Na^+^, which are generally considered incompatible with (nano-)electrospray mass spectrometry. Recently, Williams et al. have demonstrated an approach, based on the use of sub-μM ESI emitters, that seems to allow observation of native-like charge state distributions from a range of non-volatile buffers [44–47]. There have been concerns that use of these emitters leads to significant interaction between the protein and the negatively charged glass surface [46] as well as a decrease in pH at the emitter tip [48], possibly causing denaturation [48, 49]. Furthermore, as a stable spray over the time scale of the experiment—typically ca. 45 min, up to several hours in some cases—was needed for tandem MS, we wanted to avoid the previously acknowledged clogging issues associated with these ultrafine emitters [45], especially as these issues are likely to be exacerbated when spraying an aggregation-prone peptide such as amyloid β. As such, we opted to use more conventional nano-ESI emitters (described in the “Experimental” section) when comparing different buffers.

To avoid excessive Na^+^ concentrations, a buffer was prepared based on ammonium acetate and ammonium phosphate, and the pH was adjusted to 7.4 by dropwise addition of 35% aqueous NH_3_. Similarly, an analog for Krebs-Henseleit (KH) buffer, which we have used in previous—synchrotron-based—studies of amyloid/metal interactions [50, 51] was made based on ammonium acetate and piperazine-N,N′-bis(2-ethanesulfonic acid) (PIPES). Performance of these buffers was compared to pure water and aqueous ammonium acetate. While some peptide signal was detected using this phosphate-containing buffer, signal intensity was low and significant adduct formation of the peptide with H_3_PO_4_ clusters was observed (data not shown). In the KH buffer analog, no peptide signal was detected and it was found that PIPES even at a concentration as low as 1 mM completely suppressed peptide ionization. The use of pure H_2_O led to very good signal intensity; however, significant peptide binding to trace amounts of Na^+^ was observed, whereas this was mostly abolished by adding ammonium acetate. As a result, it was decided to carry out the rest of these experiments using 25 mM aqueous ammonium acetate. It could be questioned as to what extent results obtained in (non-physiological) ammonium acetate buffer are relevant for understanding the peptide’s normal behavior. To verify that amyloid β (1-42) did not lose its capability to aggregate under these conditions, peptide solutions were prepared in aqueous ammonium acetate, pure H_2_O, and KH buffer, and incubated for 375 h (approximately 15 days) at 37 °C. Subsequent TEM imaging of these samples in all cases revealed the typical fibril networks expected for this peptide (see Supporting Information S1).

### Formation of amyloid-metal complexes was straightforward in most cases, but Fe^3+^ required stabilization toward hydrolysis and aggregation by a chelating agent at physiological pH

For mass spectrometry, we initially added 12.5 μM of metal salt to the peptide solution, to provide a 1:1 molar ratio, and measured the degree of adduct formation with native MS. This way, abundant adduct formation with Cu^2+^, Ni^2+^, and Co^2+^ was observed (see Supporting Information S2). The other metals showed no or only limited binding under these conditions, so their concentration was increased to 125 μM (i.e., a metal:peptide molar ratio of 10:1), leading to significant adduct formation in most cases (Supporting Information S2). For Na^+^ and K^+^, adduct formation was still quite limited even under these conditions, likely a result of these alkali metals competing for binding sites with the far more abundant (25 mM) singly charged NH_4_^+^ ion from ammonium acetate. The fact that adducts with the NH_4_^+^ ions were not observed is easily explained by the facile loss of NH_3_ in the gas phase [52]. As addition of NaCl or KCl did not significantly affect the solution pH, it was decided to test binding of these cations in pure H_2_O, without addition of ammonium acetate. Under these conditions, sufficient binding for tandem MS was observed at a metal cation concentration of 12.5 μM (Supporting Information S2). Based on their ability to bind most of the available peptide in solution when added in a 1:1 ratio, even in the presence of ammonium acetate, we conclude that, of the metals tested here, Cu^2+^, Ni^2+^, and Co^2+^ bind most strongly to amyloid β (1-42).

**Figure 1:**
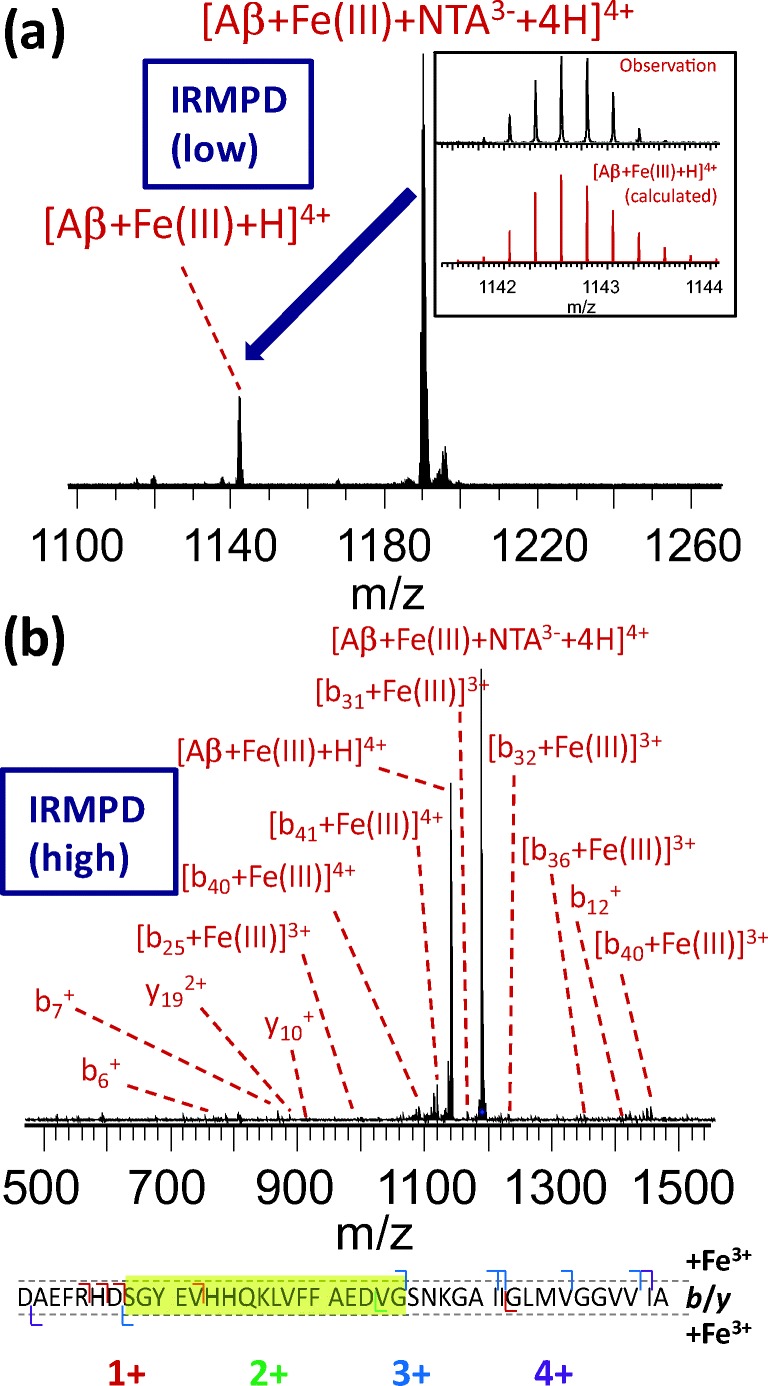
(**a**) Formation of [Aβ + Fe^3+^ + H]^4+^ after IR activation of the ternary complex between amyloid β and [Fe-NTA]. Inset shows the observed and calculated isotope distribution of the product ion, confirming the 3+ oxidation state of Fe. Longer exposure to the IR laser induced backbone fragmentation, as shown in (**b**). The observed *b*/*y* fragment ions both with and without bound Fe^3+^ are summarized below the fragment spectrum, and the apparent binding region is indicated with a yellow highlight. Fragment charge states are indicated using a color code

Increasing the FeCl_3_ concentration or incubation time did not lead to any observed binding of Fe^3+^ to the peptide, in agreement with previous work by Messori et al. [53]. The most likely explanation for this is the hydrolysis and acidity of aqua adducts, and aggregation and precipitation of bridged hydroxide/oxo species leading to poor solubility of this ion at physiological pH. To overcome this issue, we solubilized the Fe^3+^ using a chelating agent, an approach which has been successfully applied previously [54–56]. Briefly, nitrilotriacetic acid (H_3_NTA) was added to an aqueous 10 mM solution of FeCl_3_ in order to give a 1:1 mol ratio. The pH was subsequently adjusted to 7.4 by dropwise addition of 1 M aqueous NH_3_. Under these conditions, nitrilotriacetate (NTA^3-^) can act as a tetradentate ligand, with H_2_O and OH^−^ coordinating to the Fe^3+^ center, bringing the total coordination number to six [57]. This resulted in a clear solution with no visible precipitate, which was added to the 12.5 μM amyloid β solution. As was done for the other metals, we initially chose our dilution to obtain a 1:1 ratio of amyloid to the [Fe-NTA] complex. As this resulted in only very limited observation of the ternary [amyloid-Fe-NTA] complex, we increased the iron-NTA concentration to 500 μM, corresponding to a 40:1 iron:peptide ratio. This resulted in the observation of a sufficiently intense [Aβ + Fe^3+^ + NTA^3−^ + 4H]^4+^ signal to allow interrogation by tandem MS (see Supporting Information S3). No mass increase due to H_2_O or OH^−^ was observed in this complex; therefore, it seems likely that these were replaced by side chains with a free electron pair. Activation of the ternary complex using either collisions with argon atoms or irradiation by infrared laser resulted in the loss of neutral H_3_NTA and the observation of [Aβ + Fe + H]^4+^ (Figure 1). The fact that the chelating agent is lost as a protonated species under these conditions is perhaps surprising; however, loss of a 3− anion from a 7+ cation is likely to be energetically unfavorable. It has been shown previously that multiply charged protein cations can act as surprisingly strong proton donors in the gas phase due to electrostatic repulsion between protons [58]. Interestingly, further activation of this ion resulted in backbone cleavage rather than the observation of any iron-free amyloid (*vide infra*), indicating that the gas-phase interaction between Fe^3+^ and the amyloid was strong, consistent with our hypothesis that solubility was the limiting factor in the initial experiments. It has been suggested in the past that ferritin—a water-soluble protein complex and the primary Fe^3+^ reservoir in the brain—might be the origin of the mixed oxidation state iron phases that have been identified in plaque core material extracted from Alzheimer’s disease brain tissue [33, 59]. Therefore, improved understanding of the interaction between amyloid β (1-42) and Fe^3+^ could provide insight into an important event in the etiology of Alzheimer’s disease.

As shown in Supporting Information S3, despite our attempts to eliminate sodium contamination from these experiments, a few Na^+^-related “satellite” peaks were observed, likely due to the presence of trace amounts of Na^+^ in the H_3_NTA. Using the quadrupole mass filter of the solariX instrument, isolation of the [Aβ + Fe^3+^ + NTA^3−^ + 4H]^4+^ ion with a sufficiently narrow window (approximately 4 *m*/*z*) to eliminate these satellite signals resulted in a significant decrease in the precursor ion signal, complicating tandem MS experiments. As a result, in-cell isolation using CHEF was performed, resulting in near-complete elimination of Na^+^-related signals with minimal signal loss (see Supporting Information S3). As collisional activation within the ICR cell is not trivial, subsequent ion activation was performed using IRMPD. ECD of the [Aβ + Fe + H]^4+^ complex only resulted in non-dissociative charge reduction, which we attribute to the fact that three of the four protons in the peptide ion were replaced with the metal cation in this case. The IRMPD results will be discussed in detail later. First, we turn our focus toward an in-depth analysis of tandem MS results for the other amyloid-metal complexes, which were successfully interrogated with all three fragmentation methods.

### CID, ECD, and IRMPD all resulted in consistent determination of binding regions for eight (patho)physiologically relevant metals, indicating that gas-phase ligand migration did not occur to a significant extent in these experiments

For the complexes of amyloid β with Na^+^, K^+^, Mg^2+^, Ca^2+^, Mn^2+^, Co^2+^, Ni^2+^, and Cu^2+^, the 4+ charge state was isolated and subjected to fragmentation using CID (30 V collision energy), IRMPD, and ECD. For ECD, limited concomitant infrared activation [60–62] improved fragmentation efficiency without inducing the formation of the *b*/*y* ions that are characteristic for IRMPD fragmentation, and this supplemental activation was used in all cases. Regardless of the choice of fragmentation technique, numerous fragments carrying the metal were observed, providing information on the gas-phase binding region. Briefly, observation of a metal-bound N- (or C-)terminal fragment consisting of *n* residues provides strong evidence that at least one residue of the first (or last) *n* coordinates the metal ion. Conversely, observation of a metal-free fragment consisting of *m* residues can be taken as evidence that the first (or last) *m* residues do not interact strongly with the metal, although this does not necessarily hold if loss or migration of the metal is energetically more favorable than backbone cleavage and this therefore provides weaker evidence. From the above, it follows that, if multiple residues are involved in coordinating the metal cation, this can result in a “gap” where no fragmentation is observed. As metal cations are generally coordinated by several amino acid residues simultaneously, this method allows the determination of a binding region rather than detailed characterization of the metal binding site in the metal-amyloid complexes studied here.

For any given complex, the identified binding region was relatively consistent, regardless of the dissociation method used. While ECD selectively cleaves the N-Cα bond in the peptide backbone, and has therefore long been established [9, 13, 63–68] as a method for identifying noncovalent ligand binding sites, this result was somewhat unexpected for CID and IRMPD, as these are both considered “slow-heating” methods [69]. Indeed, for most noncovalent ligands, the gradual increase of internal energy, and distribution of this energy across the ion’s internal degrees of freedom, often leads to loss of these ligands prior to backbone fragmentation [64]. However, due to the ca. 80-fold lower dielectric permittivity of vacuum compared with water, electrostatic interactions were significantly strengthened, explaining why metal binding survived the CID process, while cleavage of backbone amide bonds was observed [70–73].

**Figure 2:**
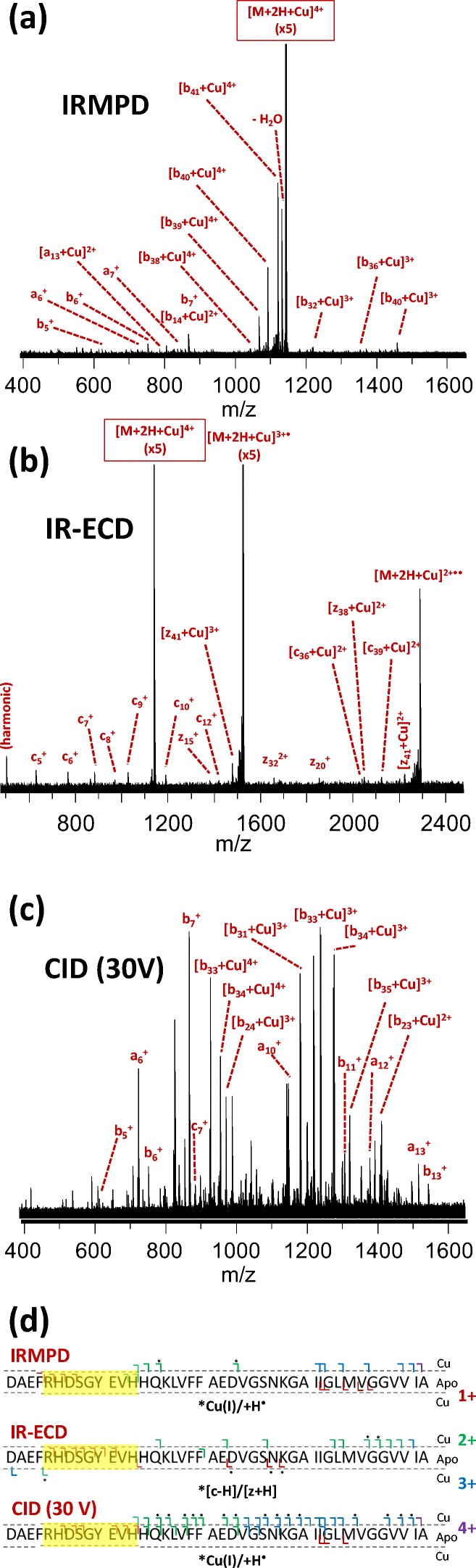
“Native top-down” fragmentation of native [Aβ + 2H + Cu]^4+^, using (**a**) IRMPD, (**b**) IR-ECD, and (**c**) CID. Fragments with and without a bound metal ion are summarized outside and inside the dotted lines in panel (**d**), respectively, and charge states are indicated using a color code. In the summary of the CID results in this panel, Cu-bound fragments with isotope distributions shifted upward by one hydrogen mass (most likely due to gas-phase reduction of Cu^2+^ to Cu^+^) are indicated with an asterisk. For IR-ECD, fragments formed through hydrogen abstraction from the *c* fragment by the corresponding *z* fragment are indicated in the same way. The “consensus” binding region that is consistent with all data obtained from the three fragmentation methods is highlighted in yellow

**Figure 3:**
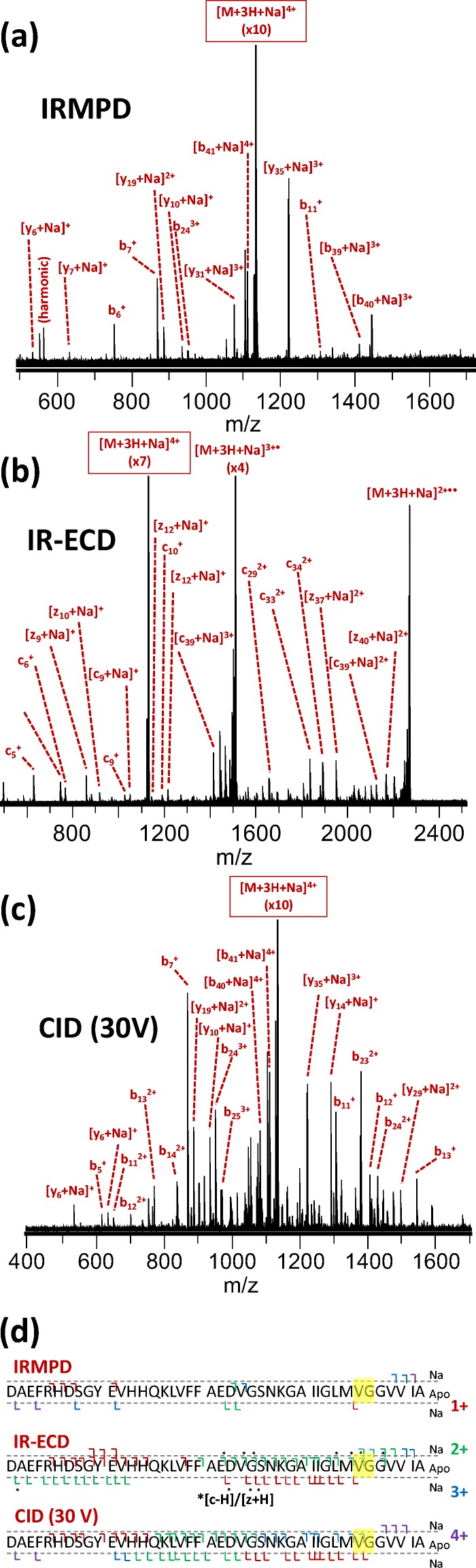
“Native top-down” fragmentation of native [Aβ + 3H + Na]^4+^, using (**a**) IRMPD, (**b**) IR-ECD, and (**c**) CID. Fragments with and without a bound metal ion are summarized outside and inside the dotted lines in panel (**d**), respectively, and charge states are indicated using a color code. For IR-ECD, fragments formed through hydrogen abstraction from the *c* fragment by the corresponding *z* fragment are indicated with an asterisk. The “consensus” binding region that is consistent with all data obtained from the three fragmentation methods is highlighted in yellow

**Figure 4:**
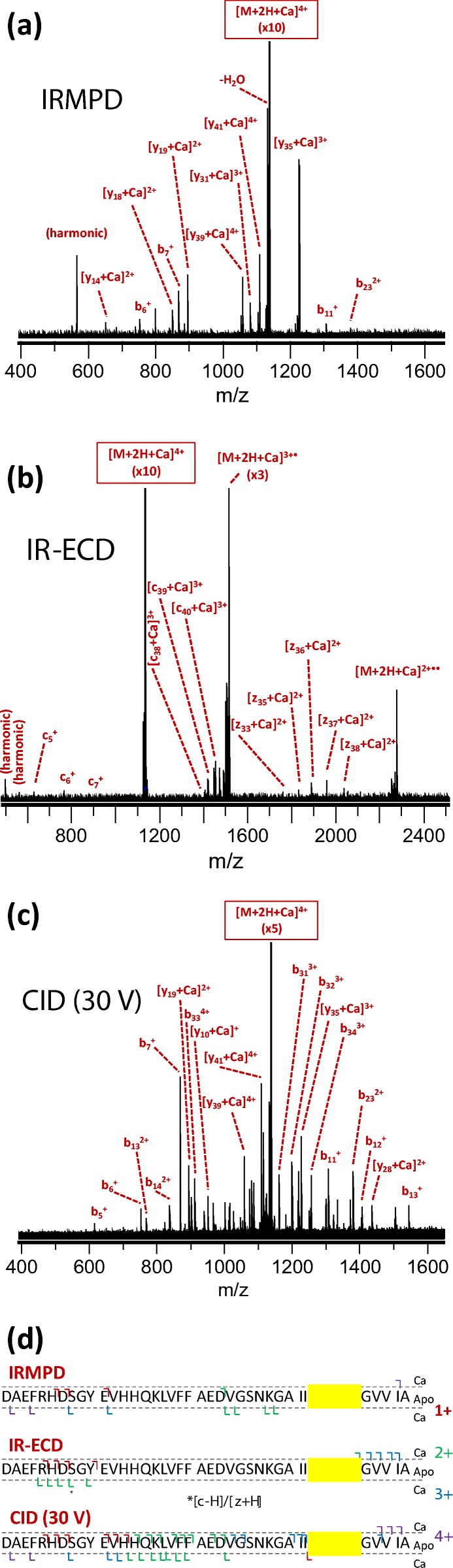
“Native top-down” fragmentation of native [Aβ + 2H + Ca]^4+^, using (**a**) IRMPD, (**b**) IR-ECD, and (**c**) CID. Fragments with and without a bound metal ion are summarized outside and inside the dotted lines in panel (**d**), respectively, and charge states are indicated with a color code. For IR-ECD, fragments formed through hydrogen abstraction from the *c* fragment by the corresponding *z* fragment are indicated with an asterisk. The “consensus” binding region that is consistent with all data obtained from the three fragmentation methods is highlighted in yellow

The identified metal binding regions for all metals are summarized in Table 1. A detailed overview of the identified fragments in tandem MS of complexes with Cu^2+^, Na^+^, and Ca^2+^ is shown in Figures 2d, 3d, and 4d, respectively. A similar overview for Co^2+^, Ni^2+^, K^+^, Mn^2+^, and Mg^2+^ can be found in Supporting Information S4. Co^2+^ and Ni^2+^ bound in a very similar region to Cu^2+^; K^+^ bound in a similar way to Na^+^; and Mn^2+^ and Mg^2+^ bound in the same region as Ca^2+^. Interestingly, the three metal ions that bound strongly at a low concentration (Ni^2+^, Cu^2+^, and Co^2+^) all did so in the N-terminal region. In IRMPD and CID of the Cu^2+^ adduct (Figure 2), all metal-bound fragments contained the [Asp1–His13] region. In these spectra, the isotope distribution of a number of copper-bound fragment peaks was shifted upward by one hydrogen mass (as indicated in Figure 2d), most likely due to gas-phase reduction of Cu^2+^ to Cu^+^. Also, a *c*_*7*_^*+*^ fragment and a small number of *a* fragments were observed. This behavior is consistent with radical-directed dissociation, which will be discussed in detail when discussing results for top-down CID of the amyloid-[Co(NH_3_)_6_] complex (*vide infra*). Details of the in vitro and ex vivo interaction between amyloid β and copper are considered in a forthcoming paper. The IR-ECD spectrum of native [Aβ + 2H + Cu]^4+^ revealed that the [Arg5–Gln15] region—which includes His6, His13, and His14—contained the Cu^2+^ binding site (Figure 2b, d). Combined, these data suggest that the metal ion was coordinated by ligands in the N-terminal region, likely including His6 and His13. Importantly, this finding matches literature results obtained using other (solution-phase) methods [30, 34]. This match, along with the remarkable consistency between CID, IRMPD, and ECD results, provides compelling evidence that the metal binding site remained more or less unchanged during ionization and transfer into the gas phase, and hence that the method used here accurately probed these sites on amyloid β.

**Table 1:**
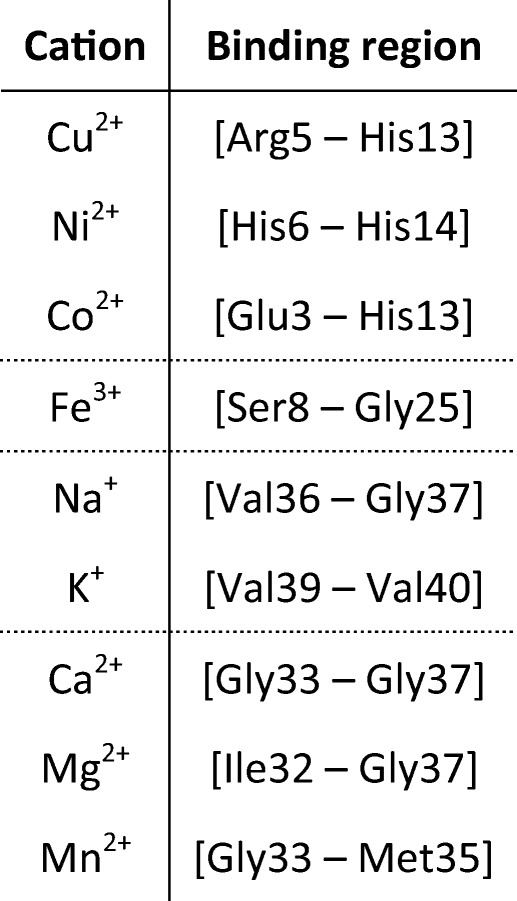
Overview of Identified Binding Regions for the Metal Cations Investigated in This Study. Dotted Lines Are Used to Divide the Metals into Groups with Similar Binding Regions

The hypothesis that several residues were involved in coordinating these metals is supported by the fragmentation of the Ni^2+^-bound peptide (Supporting Information S4). In both CID and IRMPD, a metal-bound *b*_*6*_ fragment was observed, demonstrating an interaction between the metal and (at least one of) the first six N-terminal residues of the peptide. Metal-bound *b*_*7*_ and *b*_*12*_ fragments were exclusively found in the CID spectrum, and signal intensity increased significantly for fragments larger than *b*_*12*_, which contain both His6 and His13. At the same time, the observation of metal-bound *z*_*30*_, *z*_*32*_, *z*_*33*_, and *z*_*34*_ in ECD provided evidence that binding of Ni^2+^ involves at least one of the residues in the [His13–Ala42] region. Therefore, our data indicated that Ni^2+^ is coordinated by multiple ligands, including His6 and His13. This is consistent with findings reported by Sóvágó and colleagues, who used UV-Vis and circular dichroism spectroscopy to study the complex formed between Ni^2+^ and amyloid β (1-16) in solution [34]. Similarly, for the Co^2+^ adduct, the observation of metal-bound *b*_*13*_ (CID) and *z*_*40*_ (IR-ECD) fragments restricted the possible binding site to [Glu3–His13], nearly identical to what was observed for Cu^2+^.

In contrast to the strongly binding metals discussed so far, the metals that bound more weakly to the peptide did so in the C-terminal region. Na^+^ was found to bind in the [Val36–Val39] region using IRMPD, the [Gly37–Gly38] region using CID, and at Val36 with ECD (see Table 1 and Figure 3). Similarly, K^+^ appeared to bind in the [Val39–Val40] region using a combination of the three dissociation methods (see Table 1 and Supporting Information S4). While it might be expected that these ions would bind at either a basic or a negatively charged (deprotonated) site, no polar side chains are found in the region between Val36 and Val40. As such, these ions were most likely coordinated purely by backbone amide oxygens, as has been observed previously in other peptides [74, 75]. The selectivity for this particular region was most likely due to the very high backbone flexibility and side chain hydrophobicity of this part of the peptide. The discrepancy between CID and ECD results for Na^+^ is likely due to minor variability in binding site, with multiple residues being involved as seen for Ni^2+^. Interestingly, three N-terminal ECD fragments (*c*_*9*_^*+*^, *c*_*10*_^+^, *c*_*11*_^*+*^) were observed which were bound to Na^+^. With or without addition of Na^+^, no “zIc”-type internal fragments (i.e., possessing a *z*-type N-terminus and a *c*-type C-terminus [76]) or obvious neutral losses would give rise to signals close enough to those of these *c* fragments to be misidentified. Therefore, we conclude that some—presumably very weak—interaction did in fact occur between Na^+^ and certain N-terminal residues, either to complete the coordination sphere around the metal center or as a minor, co-existing binding site. Evidently, native top-down ECD was sensitive enough to detect this minor interaction. The K^+^-bound analogs of these N-terminal fragments were not observed in ECD of [M + 3H + K]^4+^. It is also noteworthy that cleavage coverage was very good for the sodiated and potassiated peptides, similar to previous observations on small peptoids made by Ren and colleagues [77].

Based on the discussion so far, one might assume that the charge state of the metal cation determines the binding strength and site to amyloid β (1-42)—doubly charged Cu^2+^, Ni^2+^, and Co^2+^ bound easily to the N-terminal histidine residues of the peptide, while singly charged Na^+^ and K^+^ bound weakly to a site near the C-terminus. Ca^2+^, Mg^2+^, and Mn^2+^, however, also bound fairly weakly, and did so at a slightly different site compared with the alkali metals. As backbone cleavage for the adduct with Ca^2+^ was fairly limited, the binding region could not be specified further than [Gly33–Gly37] using a combination of our three dissociation methods (Figure 4). Similarly, the residues binding Mg^2+^ were located within the region [Ile32–Gly37], while the Mn^2+^ adduct showed more extensive fragmentation and the binding region in this case was narrowed down to [Gly33–Met35] (see Table 1 and Supporting Information S4). It has long been established that the Mn^2+^ ion has very similar coordination preferences to those of Mg^2+^, resulting in complexes with similar geometries [78]. Therefore, although the Mg^2+^ and Ca^2+^ binding regions could not be determined as specifically as that for Mn^2+^ in our MS experiments, it is likely that all three metals shared the same binding site. Warmlander and colleagues, based on solution NMR, concluded that Mn^2+^ binds primarily to the N-terminal histidine side chains of amyloid β (1-40) [39], although it was noted that Mn^2+^ does not compete for N-terminal histidine binding sites with Cu^2+^. Conversely, this could merely reflect the much greater stability of the Cu^2+^ complex compared with the Mn^2+^ analog [79]. These authors also identified a second binding site involving Glu22 and Asp23. Presumably, completing the coordination sphere around Mn^2+^ required involvement of backbone amide groups and we note that, as with the alkali metals Na^+^ and K^+^, the region found to bind the metal is very hydrophobic and possesses a relatively flexible structure. Recently, binding of Ca^2+^ to α-synuclein was investigated by Kaminski-Schierle and colleagues using NMR, and binding was also found to involve a relatively hydrophobic, flexible part of the peptide [80].

For all three fragmentation methods used here, fragmentation efficiency, cleavage coverage, and fragment charge states for the amyloid-metal complexes were broadly similar to those observed for the metal-free peptide (Supporting Information S5), implying that metal bridge formation did not significantly affect fragmentation. It has been observed previously that reducing the number of mobile protons generally leads to reduced fragmentation efficiency in CID, particularly for intermediate and large proteins [81, 82]. For this reason, fragmentation might be expected to be inhibited in the amyloid-metal complexes studied here. That such inhibited fragmentation was not observed is potentially due to the relatively small size of the amyloid β peptide.

### IRMPD of the ternary [Aβ-Fe-NTA] complex resulted in limited fragmentation, and indicated binding of Fe^3+^ in the [His13–Asp23] region

As noted earlier, analysis of the ternary [Aβ + Fe^3+^ + NTA^3−^ + 4H]^4+^ complex proved particularly challenging. IRMPD provided some insight, as limited activation resulted in loss of H_3_NTA and the observation of [Aβ + Fe + H]^4+^ at *m*/*z* 1142.053. Upon further irradiation, no loss of iron was observed but limited backbone fragmentation occurred (Figure 1). Based on the excellent consistency between results from CID, IRMPD, and ECD for the other eight complexes described in the previous section, it can be assumed that the IRMPD fragments accurately reflect the gas-phase structure of the [amyloid-Fe^3+^] complex. The observation of Fe^3+^-bound *b*_*22*_ and *y*_*35*_ fragments shows that the binding region is located between Ser8 and Gly25, a region containing Tyr, Glu, His, and Asp residues, all potential Fe^3+^ binding sites. Due to the complete absence of signal corresponding to iron-free peptide, the observation of metal-free fragments *b*_*12*_^*+*^ and *y*_*19*_^*2+*^ and absence of fragments spanning the [His13–Asp23] region can be seen as evidence that the main interaction region with Fe^3+^ was likely situated in this stretch of the peptide sequence. As NTA^3−^ coordinates Fe^3+^ via three acetate moieties, and its loss was coupled to the loss of three protons from the peptide, it is very likely that binding of the iron to the peptide was mediated through several residues in this region. The precise charge sites of gas-phase amyloid β (1-42) are unknown at this point, and it is known that protonation can occur at non-basic amino acid residues such as glutamine in addition to lysine, arginine, and histidine [83, 84]. Therefore, our data do not allow us at this point to ascertain whether the Fe^3+^ “replaced” three protons and was coordinated by the (formerly) protonated residues, or if proton loss and coordination of Fe^3+^ occur at different sites in a more complex rearrangement. The amount of information on the interaction of Fe^3+^ with amyloid β that is available in the literature is limited, as the poor solubility of the metal ion and aggregation of the peptide make this system highly challenging to investigate using solution-phase techniques [85]. More detailed studies of iron binding to amyloid β are therefore underway; however, we note that His13, Glu22, and Asp23 have all been suggested previously to be involved in this interaction [54, 86, 87], in good agreement with our findings.

### Gas-phase metal reduction and radical-directed dissociation were observed in CID of the amyloid-[Co(NH_3_)_6_] complex

**Figure 5:**
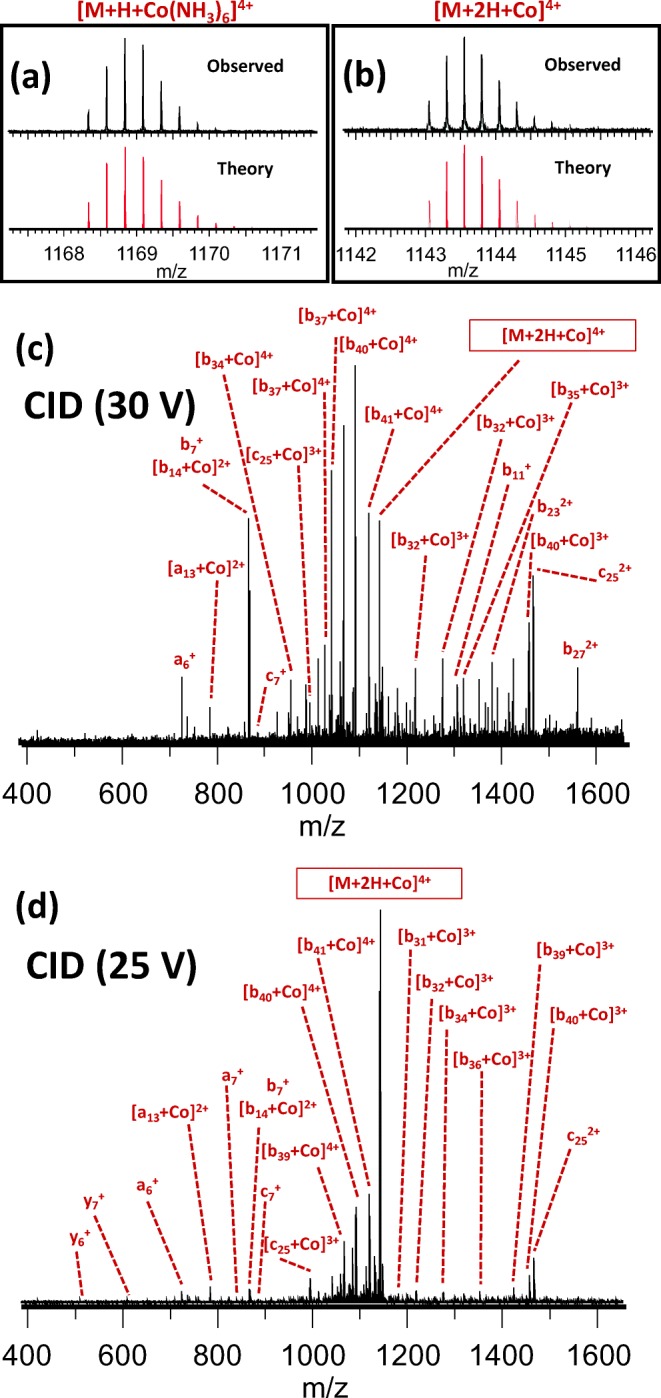
Observed versus calculated isotope distribution (**a**) for [Aβ + H + Co(NH_3_)_6_]^4+^ and (**b**) for the [Aβ + 2H + Co]^4+^ ion observed upon collisional activation of the complex; the bottom two panels show top-down fragmentation of native [Aβ + H + Co(NH_3_)_6_]^4+^, using CID at (**c**) 30 V, and (**d**) 25 V. An overview of all fragments identified in (**c**) and (**d**) can be found in Supporting Information S6

As discussed, binding of Co^2+^ involved the same N-terminal histidines as Cu^2+^ and Ni^2+^. Interesting behavior was observed when cobalt was added as [Co(NH_3_)_6_]Cl_3_, a highly stable Co^3+^ complex, rather than Co(II)Cl_2_. This complex bound to amyloid β as strongly as Ca^2+^ or Mn^2+^ (see Supporting Information S2), and the theoretical isotope distribution of [M + H + Co(NH_3_)_6_]^4+^ is compared with the observed distribution in Figure 5a. Upon CID, the NH_3_ ligands dissociated and cobalt was transferred to the N-terminus of the amyloid β peptide. Unexpectedly, rather than [M + H + Co]^4+^, the observed isotope distribution of the resulting peptide-cobalt complex matched that of [M + 2H + Co]^4+^ (Figure 5b), which indicates that, during ligand dissociation, Co^3+^ was reduced to Co^2+^. This is somewhat similar to what was observed by O’Hair and colleagues, using three different hexapeptides and [Co(salen)]OAc (where salen = N,N‘-ethylenebis(salicylideneaminato)) [88]. This process introduced a radical site in the peptide, leading to a mix of collision-induced and radical-directed dissociation (RDD; Figure 5c). The latter was evidenced by the presence of predominantly *a*-ions in addition to the *b*- and *y*-type fragments typical for CID, with *c*-ions occurring N-terminal to serine, in agreement with the RDD literature [89, 90].

An *a*-ion, by definition, is exactly one CO unit lower in mass than the corresponding *b*-ion. As such, it is also plausible that *a*-ions were formed by CO loss from a *b*-ion. It is unlikely that the *a*-ions observed in our experiments were formed this way, as they did not appear in CID spectra of the metal-free peptide, nor in CID of adducts with other metals besides [Co(NH_3_)_6_]^3+^ and Cu^2+^. To further rule out secondary fragmentation of “normal” *b*-ions as the source of the observed *a* fragments, we repeated the CID experiment using a collision energy of 25 V. As shown in Figure 5d, this still resulted in observation of both *a*- and *c*-ions, making the secondary fragmentation mechanism even more unlikely. Furthermore, while formation of *a*-ions can be explained through an even-electron mechanism (i.e., loss of CO), the presence of *c*-ions in the spectra is difficult to rationalize without invoking a radical-based mechanism. We therefore conclude that it is far more likely that these fragments were indeed formed by RDD. While radical-directed dissociation has so far only received fairly limited attention from the MS community, it should be noted that inclusion of the *a* and *c* fragments in the analysis significantly increased cleavage coverage. A great deal of excellent research on RDD of peptides and intact proteins has been performed by Julian et al., who used homolytic ultraviolet photodissociation of C–I bonds (either in a lariat crown ether [91], or formed through iodination of specific amino acid side chains [92–96]) to generate radicals. However, to the best of our knowledge, this is the first time that a relatively large peptide has been analyzed in an RDD experiment where the radical was generated through collisional activation of a peptide-metal complex, as well as the first reported use of Co(NH_3_)_6_Cl_3_ as an RDD reagent.

## Conclusions

Complexes of amyloid β (1-42) with nine bioavailable and potentially (patho)physiologically relevant metals have been studied with native FTICR mass spectrometry, with the aim of preserving the metal-peptide interaction. For each metal, the gas-phase binding region was successfully determined using tandem MS via three different activation techniques. Consistently, and owing to the low dielectric permittivity of vacuum, the metal-peptide interactions were sufficiently strong in the gas phase that determination of the binding region was possible using collision-induced dissociation, leading to results matching those from more “gentle” ECD and IRMPD. This indicates that ligand migration in the gas phase was not a major issue in tandem MS of peptide-metal complexes using any of these dissociation techniques, and that the method that is most conveniently available and/or leads to the most efficient dissociation can be used by itself to confidently identify gas-phase binding regions. Formation of an [amyloid-Fe^3+^] complex at physiological pH was observed only when a chelating agent was used to minimize aggregation and precipitation of hydroxide/oxido Fe^3+^ species, followed by mild gas-phase activation. This observation is important and has implications for the design of future studies of peptide interactions with metal ions which readily form hydroxide and oxido species at physiological pH, e.g., Al^3+^ and Zn^2+^.More extensive activation of the Fe^3+^ complex suggested interaction with amyloid β (1-42) in the region between residues His13 and Asp23.

The binding regions identified in the gas phase using tandem MS are consistent with previous studies using solution-phase methods. The metals that exhibited the strongest binding to the peptide all share a very similar binding region involving the N-terminal histidine residues. The metals studied display a strong preference for a particular region, although not the same region. This was observed even for the alkali metals Na^+^ and K^+^, which are often assumed to engage in non-specific binding in (native) mass spectrometry. Collisional activation of the complex with [Co(NH_3_)_6_]^3+^ induced unexpected gas-phase redox chemistry and radical-directed peptide dissociation, and inclusion of the resulting fragments in the data analysis significantly increased the observed cleavage coverage. The insights gained from this fast, robust MS method, into the interactions between metal cations and amyloid β, are likely to contribute to improved understanding and treatment of Alzheimer’s disease.

## Electronic supplementary material

ESM 1 (DOCX 2603 kb)
