# Supplementary material for: Metal Ion Binding to the Amyloid β Monomer Studied by Native Top-Down FTICR Mass Spectrometry
Source: J Am Soc Mass Spectrom. 2019 Jul 26;30(10):2123–34. doi: 10.1007/s13361-019-02283-7 (PMC6805827; doi:10.1007/s13361-019-02283-7)
Supplement: Supplementary file 1 — (DOCX 2603 kb) [file 13361_2019_2283_MOESM1_ESM.docx]

**Supporting Information for ‘Metal ion binding to the amyloid β monomer studied by native top-down FTICR mass spectrometry‘**

Frederik Lermyte^1,2^, James Everett^3^, Yuko P.Y. Lam^2^, Christopher A. Wootton^2^, Jake Brooks^1^, Mark P. Barrow^2^, Neil D. Telling^3^, Peter J. Sadler^2^, Peter B. O’Connor^2^, Joanna F. Collingwood^1^

^1^School of Engineering, University of Warwick, Coventry CV4 7AL, UK

^2^Department of Chemistry, University of Warwick, Coventry CV4 7AL, UK

^3^Institute for Science and Technology in Medicine, Keele University, Stoke-on-Trent, Staffordshire ST4 7QB, UK

**Figure S1:** TEM images of 35 µM amyloid β (1-42) in aqueous ammonium acetate, H_2_O, and KH buffer, after incubation at 37 degrees Celsius for 375 hours.

**Figure S2:** Native spectra of 12.5 µM amyloid β (1-42) with 12.5 or 125 µM of metal cations (a metal complex in the case of [Co(NH_3_)_6_]^3+^).

**Figure S3:** Complex formation between amyloid β and the [Fe-NTA] complex.

**Figure S4:** Summary of ‘native top-down’ tandem MS of native [Aβ + (4-n)H + Metal^n+^]^4+^, where Metal^n+^ is Ni^2+^, Co^2+^, K^+^, Mn^2+^, and Mg^2+^.

**Figure S5:** ‘Native top-down’ fragmentation of native [Aβ + 4H]^4+^, using IRMPD, IR-ECD, and CID.

**Figure S6:** Summary of ‘native top-down’ tandem MS of native [Aβ + H + Co(NH_3_)_6_]^4+^ using 25 and 30 V of CID.

**Supporting Information S7**: Fragment assignment for native top-down MS of the peptide-metal complexes investigated in this study.

**Figure S1:** TEM images of amyloid β (1-42) after incubation at 37 °C for 375 hours, at a magnification of 12000 X, using a peptide concentration of (A) 35 µM in 100 mM aqueous ammonium acetate, (B) 35 µM in Milli-Q grade H_2_O, and (C) 12.5 µM in Krebs-Henseleit buffer.

**Figure S2:** Native spectra of 12.5 µM amyloid β (1-42) with 12.5 or 125 µM of metal cations (a metal complex in the case of [Co(NH_3_)_6_]^3+^). Note that the samples with 12.5 µM Na^+^ or K^+^ did not contain any ammonium acetate, as this virtually abolished binding of the alkali metals.

**Figure S3:** (A) Complex formation between amyloid β and the [Fe-NTA] complex. Inset shows the observed *vs.* calculated isotope pattern for the 4+ adduct, showing that the iron complex was neutral overall, and all four charges were carried by protons. The other peaks between *m/z* 1170 and 1220 were due to substitution of Fe^3+^ and/or protons with Na^+^ ions in the [Ab + Fe^3+^ + NTA^3-^ + 4H]^4+^ complex. Panel (B) shows the spectrum after isolation of the complex using CHEF, but without any ion activation, virtually eliminating all signals other than the precursor of interest.

**Figure S4:** Summary of ‘native top-down’ tandem MS of native [Aβ + (4-n)H + Metal^n+^]^4+^, where Metal^n+^ is Ni^2+^, Co^2+^, K^+^, Mn^2+^, and Mg^2+^. Each panel shows the results obtained with IRMPD (top row), IR-ECD, (second row), and CID at 30 V (third row). Fragments with and without a bound metal ion are indicated outside and inside the dotted lines, respectively. The ‘consensus’ binding region that is consistent with all data obtained from the three fragmentation methods is highlighted in yellow. Fragments labelled with as asterisk show a prominent peak in their isotope distribution that has been shifted by one hydrogen mass, as indicated under each row.

**Figure S5:** ‘Native top-down’ fragmentation of native [Aβ + 4H]^4+^, using (A) IRMPD, (B) IR-ECD, and (C) CID.

**Figure S6:** Summary of ‘native top-down’ tandem MS of native [Aβ + H + Co(NH_3_)_6_]^4+^ using 25 (top panel) and 30 V (bottom panel) of CID. Fragments formed *via* different pathways are shown on separate rows, with ‘standard’ (for CID) *b*/*y* fragments on the second row, *a* fragments (likely formed via a radical pathway, but conceivably through an even-electron mechanism) on the top row, and *c* fragments (only formed by a radical mechanism) on the bottom row. As expected from existing literature on radical-directed dissociation, *c* fragments are exclusively observed adjacent to serine residues (indicated with red arrows). Fragments with and without a bound metal ion are indicated outside and inside the dotted lines, respectively. The ‘consensus’ binding region that is consistent with all data obtained from the three fragmentation methods is highlighted in yellow.

**CID (30 V) of [M+2H+Cu]^4+^**

| **Fragment** | **Exact m/z** | **Observed m/z** | **Mass error (ppm)** |
| --- | --- | --- | --- |
| [b5]+ | 619.2835 | 619.2833 | -0.3 |
| [b6]+ | 756.3424 | 756.3424 | 0.0 |
| [b7]+ | 871.3694 | 871.3693 | 0.0 |
| [b8]+ | 958.4014 | 958.4010 | -0.4 |
| [b11]+ | 1307.5288 | 1307.5278 | -0.7 |
| [b12]+ | 1406.5972 | 1406.5973 | 0.1 |
| [b13]+ | 1543.6561 | 1543.6560 | -0.1 |
| [b14]2+ | 840.8612 | 840.8609 | -0.3 |
| [b15]2+ | 904.8904 | 904.8895 | -1.0 |
| [b17]2+ | 1025.4800 | 1025.4782 | -1.8 |
| [b18]2+ | 1075.0142 | 1075.0142 | 0.0 |
| [b24]3+ | 953.1158 | 953.1155 | -0.3 |
| [b25]3+ | 972.1230 | 972.1226 | -0.4 |
| [b27]3+ | 1039.1480 | 1039.1473 | -0.7 |
| [a6]+ | 728.3475 | 728.3475 | 0.0 |
| [a7]+ | 843.3744 | 843.3745 | 0.1 |
| [a10]+ | 1150.4913 | 1150.4905 | -0.6 |
| [a12]+ | 1378.6023 | 1378.6026 | 0.3 |
| [a13]+ | 1515.6612 | 1515.6612 | 0.0 |
| [a13]2+ | 758.3342 | 758.3346 | 0.4 |
| [a14]2+ | 826.8637 | 826.8645 | 1.0 |
| [c7]+ | 888.3959 | 888.3958 | -0.1 |
| [y8]+ | 745.4277 | 745.4273 | -0.5 |
| [y10]+ | 915.5332 | 915.5325 | -0.8 |
| [y11]+ | 1028.6173 | 1028.6164 | -0.9 |
| [b15+Cu(I)]2+ | 935.8513 | 935.8520 | 0.7 |
| [b16+Cu(I)]2+ | 999.8988 | 999.8988 | 0.0 |
| [b18+Cu(I)]2+ | 1105.9750 | 1105.9757 | 0.6 |
| [b19+Cu(I)]2+ | 1179.5092 | 1179.5101 | 0.8 |
| [b20+Cu(I)]2+ | 1253.0435 | 1253.0436 | 0.1 |
| [b22+Cu(I)]2+ | 1353.0833 | 1353.0835 | 0.1 |
| [b23+Cu(I)]2+ | 1410.5968 | 1410.5960 | -0.6 |
| [b26+Cu(I)]3+ | 1021.7743 | 1021.7753 | 1.0 |
| [b27+Cu(I)]3+ | 1059.7886 | 1059.7884 | -0.2 |
| [b28+Cu(I)]3+ | 1102.4869 | 1102.4861 | -0.7 |
| [b29+Cu(I)]3+ | 1121.4940 | 1121.4949 | 0.8 |
| [b35+Cu(I)]3+ | 1320.9445 | 1320.9435 | -0.7 |
| [b38+Cu(I)]3+ | 1391.9816 | 1391.9797 | -1.4 |
| [b39+Cu(I)]3+ | 1425.0044 | 1425.0030 | -0.9 |
| [b40+Cu(I)]3+ | 1458.0272 | 1458.0257 | -1.0 |
| [a13+Cu(I)]2+ | 789.2951 | 789.2951 | -0.1 |
| [a14+Cu(I)]2+ | 857.8246 | 857.8248 | 0.3 |
| [a15+Cu(I)]2+ | 921.8539 | 921.8533 | -0.6 |
| [a18+Cu(I)]2+ | 1091.9776 | 1091.9784 | 0.7 |
| [a19+Cu(I)]2+ | 1165.5118 | 1165.5113 | -0.4 |
| [a20+Cu(I)]2+ | 1239.0460 | 1239.0455 | -0.4 |
| [a24+Cu(I)]3+ | 964.4248 | 964.4248 | 0.1 |
| [a27+Cu(I)]3+ | 1050.4569 | 1050.4561 | -0.8 |
| [a31+Cu(I)]3+ | 1173.5361 | 1173.5360 | -0.1 |
| [a32+Cu(I)]3+ | 1211.2308 | 1211.2310 | 0.2 |
| [a33+Cu(I)]4+ | 922.9303 | 922.9294 | -1.0 |
| [b13+Cu(II)]2+ | 802.7887 | 802.7884 | -0.3 |
| [b14+Cu(II)]2+ | 871.3181 | 871.3178 | -0.4 |
| [b24+Cu(II)]3+ | 973.4205 | 973.4197 | -0.8 |
| [b25+Cu(II)]3+ | 992.4276 | 992.4286 | 0.9 |
| [b30+Cu(II)]3+ | 1144.8371 | 1144.8410 | 3.4 |
| [b31+Cu(II)]3+ | 1182.5318 | 1182.5308 | -0.9 |
| [b32+Cu(II)]3+ | 1220.2265 | 1220.2262 | -0.2 |
| [b33+Cu(II)]3+ | 1239.2337 | 1239.2330 | -0.5 |
| [b34+Cu(II)]3+ | 1276.9283 | 1276.9283 | 0.0 |
| [b31+Cu(II)]4+ | 887.1507 | 887.1511 | 0.4 |
| [b32+Cu(II)]4+ | 915.4217 | 915.4220 | 0.4 |
| [b33+Cu(II)]4+ | 929.6771 | 929.6772 | 0.1 |
| [b34+Cu(II)]4+ | 957.9481 | 957.9473 | -0.8 |
| [b35+Cu(II)]4+ | 990.7082 | 990.7079 | -0.3 |
| [b36+Cu(II)]4+ | 1015.4753 | 1015.4771 | 1.8 |
| [b37+Cu(II)]4+ | 1029.7307 | 1029.7299 | -0.7 |
| [b38+Cu(II)]4+ | 1043.9860 | 1043.9856 | -0.4 |
| [b39+Cu(II)]4+ | 1068.7531 | 1068.7520 | -1.1 |
| [b41+Cu(II)]4+ | 1121.7913 | 1121.7920 | 0.6 |
| Average |  |  | -0.13 |
| St Dev |  |  | 0.76 |

**IRMPD of [M+2H+Cu]^4+^**

| **Fragment** | **Exact m/z** | **Observed m/z** | **Mass error (ppm)** |
| --- | --- | --- | --- |
| [b4]+ | 463.1824 | 463.1834 | 2.2 |
| [b5]+ | 619.2835 | 619.2835 | 0.0 |
| [b6]+ | 756.3424 | 756.3425 | 0.1 |
| [b7]+ | 871.3694 | 871.3694 | 0.0 |
| [b12]2+ | 703.8022 | 703.8026 | 0.5 |
| [b13]2+ | 772.3317 | 772.3319 | 0.3 |
| [a6]+ | 728.3475 | 728.3476 | 0.1 |
| [a7]+ | 843.3744 | 843.3739 | -0.6 |
| [y6]+ | 515.3188 | 515.3195 | 1.2 |
| [y7]+ | 614.3872 | 614.3882 | 1.5 |
| [y8]+ | 745.4277 | 745.4283 | 0.7 |
| [y10]+ | 915.5332 | 915.5333 | 0.0 |
| [y11]+ | 1028.6173 | 1028.6175 | 0.2 |
| [b15+Cu(I)]2+ | 935.8513 | 935.8507 | -0.7 |
| [b23+Cu(I)]2+ | 1410.5968 | 1410.5943 | -1.7 |
| [b23+Cu(I)]3+ | 940.7336 | 940.7326 | -1.1 |
| [b38+Cu(I)]4+ | 1044.2380 | 1044.2381 | 0.1 |
| [b14+Cu(I)+H]2+ | 789.7988 | 789.7997 | 1.2 |
| [b39+Cu(I)]4+ | 1062.0064 | 1062.0050 | -1.3 |
| [b40+Cu(I)]4+ | 1086.7735 | 1086.7734 | -0.1 |
| [b42+Cu(I)+H]4+ | 1133.0556 | 1133.0560 | 0.4 |
| [b13+Cu(I)]2+ | 802.7887 | 802.7895 | 1.0 |
| [b14+Cu(I)]2+ | 871.3181 | 871.3184 | 0.4 |
| [b31+Cu(I)]3+ | 1182.5318 | 1182.5338 | 1.6 |
| [b32+Cu(I)]3+ | 1220.2265 | 1220.2271 | 0.5 |
| [b34+Cu(I)]3+ | 1276.9283 | 1276.9281 | -0.2 |
| [b36+Cu(I)]3+ | 1353.6313 | 1353.6299 | -1.0 |
| [b39+Cu(I)]3+ | 1424.6684 | 1424.6682 | -0.1 |
| [b40+Cu(I)]3+ | 1457.6912 | 1457.6925 | 0.8 |
| [b39+Cu(I)]4+ | 1068.7531 | 1068.7532 | 0.1 |
| [b40+Cu(I)]4+ | 1093.5202 | 1093.5200 | -0.3 |
| [b41+Cu(I)]4+ | 1121.7913 | 1121.7914 | 0.2 |
| Average |  |  | 0.19 |
| St Dev |  |  | 0.85 |

**IR-ECD of [M+2H+Cu]^4+^**

| **Fragment** | **Exact m/z** | **Observed m/z** | **Mass error (ppm)** |
| --- | --- | --- | --- |
| [b7]+ | 871.3694 | 871.3697 | 0.3 |
| [c5]+ | 636.3100 | 636.3102 | 0.2 |
| [c6]+ | 773.3690 | 773.3690 | 0.1 |
| [c7]+ | 888.3959 | 888.3963 | 0.4 |
| [c8]+ | 975.4279 | 975.4284 | 0.5 |
| [c9]+ | 1032.4494 | 1032.4497 | 0.3 |
| [c10]+ | 1195.5127 | 1195.5134 | 0.6 |
| [c11]+ | 1324.5553 | 1324.5566 | 1.0 |
| [z15+H]+ | 1382.8441 | 1382.8440 | 0.0 |
| [z16+H]+ | 1496.8870 | 1496.8850 | -1.3 |
| [z20+H]+ | 1855.0358 | 1855.0366 | 0.4 |
| [z29]2+ | 1477.3087 | 1477.3080 | -0.4 |
| [c15+Cu(I)]2+ | 944.3646 | 944.3643 | -0.3 |
| [c22+Cu(I)]2+ | 1361.5966 | 1361.5957 | -0.6 |
| [c26+Cu(I)]2+ | 1540.6710 | 1540.6700 | -0.6 |
| [c33+Cu(I)]2+ | 1867.3640 | 1867.3600 | -2.1 |
| [c34+Cu(I)]2+ | 1923.9061 | 1923.9035 | -1.3 |
| [c36+Cu(I)-H]2+ | 2038.4569 | 2038.4605 | 1.8 |
| [c37+Cu(I)-H]2+ | 2066.9676 | 2066.9691 | 0.7 |
| [c38+Cu(I)]2+ | 2095.9820 | 2095.9837 | 0.8 |
| [c39+Cu(I)]2+ | 2145.5162 | 2145.5170 | 0.4 |
| [c40+Cu(I)]3+ | 1463.7027 | 1463.7009 | -1.2 |
| [z38+Cu(I)+H]2+ | 2049.0100 | 2049.0104 | 0.2 |
| [z41+Cu(I)]3+ | 1481.7227 | 1481.7199 | -1.9 |
| Average |  |  | -0.08 |
| St Dev |  |  | 0.94 |

**CID (30 V) of [M+3H+K]^4+^**

| **Fragment** | **Exact m/z** | **Observed m/z** | **Mass error (ppm)** |
| --- | --- | --- | --- |
| [b5]+ | 619.2835 | 619.2839 | 0.6 |
| [b6]+ | 756.3424 | 756.3423 | -0.2 |
| [b7]+ | 871.3694 | 871.3698 | 0.5 |
| [b11]+ | 1307.5288 | 1307.5280 | -0.6 |
| [b12]+ | 1406.5972 | 1406.5976 | 0.3 |
| [b13]+ | 1543.6561 | 1543.6564 | 0.2 |
| [b13]2+ | 772.3317 | 772.3318 | 0.1 |
| [b14]2+ | 840.8612 | 840.8613 | 0.2 |
| [b15]2+ | 904.8904 | 904.8914 | 1.0 |
| [b16]2+ | 968.9379 | 968.9377 | -0.2 |
| [b18]2+ | 1075.0142 | 1075.0141 | 0.0 |
| [b20]2+ | 1222.0826 | 1222.0822 | -0.3 |
| [b22]2+ | 1322.1224 | 1322.1230 | 0.4 |
| [b23]2+ | 1379.6359 | 1379.6358 | -0.1 |
| [b23]3+ | 920.0930 | 920.0931 | 0.1 |
| [b24]3+ | 953.1158 | 953.1159 | 0.0 |
| [b25]3+ | 972.1230 | 972.1232 | 0.3 |
| [b26]3+ | 1001.1337 | 1001.1333 | -0.4 |
| [b31]3+ | 1162.2272 | 1162.2261 | -1.0 |
| [b32]3+ | 1199.9219 | 1199.9232 | 1.1 |
| [b33]3+ | 1218.9290 | 1218.9285 | -0.5 |
| [b34]3+ | 1256.6237 | 1256.6244 | 0.5 |
| [b40+K]4+ | 1087.7807 | 1087.7804 | -0.3 |
| [b41+K]4+ | 1116.0518 | 1116.0513 | -0.4 |
| [b42+K]4+ | 1133.8110 | 1133.8097 | -1.2 |
| [y6+K]+ | 553.2747 | 553.2746 | -0.2 |
| [y8+K]+ | 783.3836 | 783.3836 | 0.0 |
| [y10+K]+ | 953.4891 | 953.4887 | -0.4 |
| [y11+K]+ | 1066.5732 | 1066.5727 | -0.5 |
| [y12+K]+ | 1179.6573 | 1179.6576 | 0.3 |
| [y14+K]+ | 1307.7158 | 1307.7160 | 0.2 |
| [y19+K]2+ | 896.9915 | 896.9911 | -0.5 |
| [y27+K]2+ | 1371.7369 | 1371.7364 | -0.4 |
| [y29+K]3+ | 1003.1995 | 1003.1995 | -0.1 |
| [y31+K]3+ | 1081.9087 | 1081.9084 | -0.2 |
| [y35+K]3+ | 1227.2951 | 1227.2945 | -0.5 |
| [y39+K]4+ | 1059.5370 | 1059.5377 | 0.6 |
| [y41+K]4+ | 1109.5569 | 1109.5561 | -0.7 |
| Average |  |  | -0.06 |
| St Dev |  |  | 0.50 |

**IRMPD of [M+3H+K]^4+^**

| **Fragment** | **Exact m/z** | **Observed m/z** | **Mass error (ppm)** |
| --- | --- | --- | --- |
| [b5]+ | 619.28349 | 619.2835 | 0.0 |
| [b6]+ | 756.34241 | 756.34235 | -0.1 |
| [b7]+ | 871.36935 | 871.36943 | 0.1 |
| [b11]+ | 1307.52876 | 1307.52873 | 0.0 |
| [b22]2+ | 1322.1224 | 1322.1223 | -0.1 |
| [b23]2+ | 1379.6359 | 1379.6372 | 0.9 |
| [b41+K]4+ | 1116.0518 | 1116.0521 | 0.3 |
| [b42+K]4+ | 1133.8110 | 1133.8126 | 1.4 |
| [y5+K]+ | 496.2532 | 496.2532 | 0.0 |
| [y6+K]+ | 553.2747 | 553.2747 | 0.0 |
| [y7+K]+ | 652.3431 | 652.3429 | -0.3 |
| [y8+K]+ | 783.3836 | 783.3837 | 0.1 |
| [y10+K]+ | 953.4891 | 953.4895 | 0.4 |
| [y11+K]+ | 1066.5732 | 1066.5726 | -0.6 |
| [y14+K]+ | 1307.7158 | 1307.7140 | -1.4 |
| [y19+K]2+ | 896.9915 | 896.9911 | -0.4 |
| [y31+K]3+ | 1081.9087 | 1081.9073 | -1.2 |
| [y35+K]3+ | 1227.2951 | 1227.2954 | 0.2 |
| [y41+K]4+ | 1109.5569 | 1109.5580 | 0.9 |
| [y42+K]4+ | 1138.3137 | 1138.3142 | 0.4 |
| Average |  |  | 0.03 |
| St Dev |  |  | 0.65 |

**IR-ECD of [M+3H+K]^4+^**

| **Fragment** | **Exact m/z** | **Observed m/z** | **Mass error (ppm)** |
| --- | --- | --- | --- |
| [c5]+ | 636.31004 | 636.31007 | 0.0 |
| [c6]+ | 773.36896 | 773.36891 | -0.1 |
| [c7]+ | 888.3959 | 888.39594 | 0.0 |
| [c8]+ | 975.42793 | 975.42759 | -0.3 |
| [c9]+ | 1032.4494 | 1032.4492 | -0.2 |
| [c10]+ | 1195.5127 | 1195.5133 | 0.5 |
| [c11]+ | 1324.5553 | 1324.5538 | -1.2 |
| [c22]2+ | 1330.6357 | 1330.6370 | 0.9 |
| [c24]2+ | 1437.6834 | 1437.6813 | -1.5 |
| [c27]2+ | 1566.7316 | 1566.7333 | 1.1 |
| [c28]2+ | 1630.7791 | 1630.7810 | 1.2 |
| [c29]2+ | 1659.2898 | 1659.2918 | 1.2 |
| [c31]2+ | 1751.3504 | 1751.3524 | 1.2 |
| [c34]2+ | 1892.9452 | 1892.9479 | 1.4 |
| [c36]2+ | 2007.9996 | 2007.9955 | -2.0 |
| [c38]2+ | 2065.0211 | 2065.0245 | 1.7 |
| [z4+K+H]+ | 424.2209 | 424.2216 | 1.5 |
| [z5+K+H]+ | 481.2424 | 481.2427 | 0.7 |
| [z6+K+H]+ | 538.2639 | 538.2640 | 0.2 |
| [z8+K]+ | 767.3654 | 767.3650 | -0.6 |
| [z9+K]+ | 880.4495 | 880.4486 | -1.0 |
| [z10+K]+ | 937.4710 | 937.4704 | -0.6 |
| [z11+K]+ | 1050.5550 | 1050.5541 | -0.9 |
| [z12+K]+ | 1163.6391 | 1163.6377 | -1.2 |
| [z13+K]+ | 1234.6762 | 1234.6767 | 0.4 |
| [z14+K]+ | 1291.6977 | 1291.6988 | 0.9 |
| [z15+K]+ | 1419.7926 | 1419.7927 | 0.0 |
| [z17+K]+ | 1620.8676 | 1620.8669 | -0.4 |
| [z30+K+H]2+ | 1565.3197 | 1565.3192 | -0.3 |
| [z33+K]2+ | 1760.4032 | 1760.4054 | 1.3 |
| [z34+K]2+ | 1788.9139 | 1788.9156 | 0.9 |
| [z35+K]2+ | 1832.4300 | 1832.4283 | -0.9 |
| [z36+K]2+ | 1889.9434 | 1889.9419 | -0.8 |
| [z37+K]2+ | 1958.4729 | 1958.4759 | 1.5 |
| Average |  |  | 0.14 |
| St Dev |  |  | 1.00 |

**CID (30 V) of [M+3H+Na]^4+^**

| **Fragment** | **Exact m/z** | **Observed m/z** | **Mass error (ppm)** |
| --- | --- | --- | --- |
| [b5]+ | 619.2835 | 619.2840 | 0.8 |
| [b6]+ | 756.3424 | 756.3424 | 0.0 |
| [b7]+ | 871.3694 | 871.3693 | -0.1 |
| [b8]+ | 958.4014 | 958.4004 | -1.1 |
| [b10]+ | 1178.4862 | 1178.4866 | 0.4 |
| [b11]+ | 1307.5288 | 1307.5288 | 0.0 |
| [b12]+ | 1406.5972 | 1406.5975 | 0.2 |
| [b13]+ | 1543.6561 | 1543.6558 | -0.2 |
| [b14]+ | 1680.7150 | 1680.7139 | -0.6 |
| [b11]2+ | 654.2680 | 654.2683 | 0.4 |
| [b12]2+ | 703.8022 | 703.8021 | -0.2 |
| [b13]2+ | 772.3317 | 772.3318 | 0.2 |
| [b14]2+ | 840.8612 | 840.8614 | 0.3 |
| [b15]2+ | 904.8904 | 904.8906 | 0.2 |
| [b16]2+ | 968.9379 | 968.9379 | 0.0 |
| [b17]2+ | 1025.4800 | 1025.4810 | 1.0 |
| [b18]2+ | 1075.0142 | 1075.0141 | 0.0 |
| [b19]2+ | 1148.5484 | 1148.5485 | 0.1 |
| [b20]2+ | 1222.0826 | 1222.0804 | -1.8 |
| [b21]2+ | 1257.6011 | 1257.6014 | 0.2 |
| [b22]2+ | 1322.1224 | 1322.1219 | -0.4 |
| [b23]2+ | 1379.6359 | 1379.6357 | -0.1 |
| [b24]2+ | 1429.1701 | 1429.1695 | -0.4 |
| [b25]2+ | 1457.6808 | 1457.6805 | -0.2 |
| [b20]3+ | 815.0575 | 815.0576 | 0.1 |
| [b21]3+ | 838.7365 | 838.7364 | -0.1 |
| [b22]3+ | 881.7507 | 881.7507 | 0.0 |
| [b23]3+ | 920.0930 | 920.0930 | -0.1 |
| [b24]3+ | 953.1158 | 953.1159 | 0.1 |
| [b25]3+ | 972.1230 | 972.1229 | -0.1 |
| [b26]3+ | 1001.1337 | 1001.1340 | 0.3 |
| [b27]3+ | 1039.1480 | 1039.1484 | 0.4 |
| [b28]3+ | 1081.8463 | 1081.8463 | 0.0 |
| [b30]3+ | 1124.5325 | 1124.5325 | 0.0 |
| [b31]3+ | 1162.2272 | 1162.2266 | -0.5 |
| [b32]3+ | 1199.9219 | 1199.9216 | -0.3 |
| [b34]3+ | 1256.6237 | 1256.6251 | 1.1 |
| [b35]3+ | 1300.3039 | 1300.3050 | 0.8 |
| [b36]3+ | 1333.3267 | 1333.3259 | -0.6 |
| [b39+Na]4+ | 1059.0201 | 1059.0199 | -0.2 |
| [b40+Na]4+ | 1083.7872 | 1083.7873 | 0.0 |
| [b41+Na]4+ | 1112.0583 | 1112.0592 | 0.8 |
| [y6+Na]+ | 537.3007 | 537.3011 | 0.6 |
| [y7+Na]+ | 636.3692 | 636.3693 | 0.3 |
| [y8+Na]+ | 767.4096 | 767.4095 | -0.2 |
| [y9+Na]+ | 880.4937 | 880.4938 | 0.1 |
| [y10+Na]+ | 937.5152 | 937.5153 | 0.1 |
| [y11+Na]+ | 1050.5992 | 1050.5995 | 0.3 |
| [y12+Na]+ | 1163.6833 | 1163.6834 | 0.1 |
| [y13+Na]+ | 1234.7204 | 1234.7207 | 0.2 |
| [y14+Na]+ | 1291.7419 | 1291.7419 | 0.0 |
| [y16+Na]+ | 1533.8798 | 1533.8808 | 0.7 |
| [y17+Na]+ | 1620.9118 | 1620.9130 | 0.7 |
| [y18+Na]+ | 1677.9333 | 1677.9316 | -1.0 |
| [y17+Na]2+ | 810.9595 | 810.9588 | -0.9 |
| [y18+Na]2+ | 839.4703 | 839.4698 | -0.5 |
| [y19+Na]2+ | 889.0045 | 889.0047 | 0.3 |
| [y20+Na]2+ | 946.5180 | 946.5176 | -0.3 |
| [y22+Na]2+ | 1046.5578 | 1046.5588 | 1.0 |
| [y23+Na]2+ | 1120.0920 | 1120.0914 | -0.5 |
| [y24+Na]2+ | 1193.6262 | 1193.6252 | -0.8 |
| [y25+Na]2+ | 1243.1604 | 1243.1599 | -0.4 |
| [y26+Na]2+ | 1299.7025 | 1299.7009 | -1.2 |
| [y27+Na]2+ | 1363.7499 | 1363.7499 | 0.0 |
| [y28+Na]2+ | 1427.7792 | 1427.7801 | 0.6 |
| [y29+Na]2+ | 1496.3087 | 1496.3080 | -0.4 |
| [y29+Na]3+ | 997.8749 | 997.8755 | 0.6 |
| [y30+Na]3+ | 1043.5612 | 1043.5612 | 0.0 |
| [y31+Na]3+ | 1076.5840 | 1076.5840 | 0.0 |
| [y35+Na]3+ | 1221.9705 | 1221.9702 | -0.3 |
| [y37+Na]3+ | 1305.9991 | 1305.9987 | -0.3 |
| [y39+Na]4+ | 1055.5435 | 1055.5441 | 0.6 |
| [y41+Na]4+ | 1105.5634 | 1105.5628 | -0.6 |
| [y42+Na]4+ | 1134.3202 | 1134.3201 | 0.0 |
| Average |  |  | -0.01 |
| St Dev |  |  | 0.53 |

**IRMPD of [M+3H+Na]^4+^**

| **Fragment** | **Exact m/z** | **Observed m/z** | **Mass error (ppm)** |
| --- | --- | --- | --- |
| [b5]+ | 619.2835 | 619.2822 | -2.0 |
| [b6]+ | 756.3424 | 756.3423 | -0.2 |
| [b7]+ | 871.3694 | 871.3697 | 0.4 |
| [b11]+ | 1307.5288 | 1307.5264 | -1.8 |
| [b23]2+ | 1379.6359 | 1379.6353 | -0.5 |
| [b24]3+ | 953.1158 | 953.1159 | 0.0 |
| [b39+Na]3+ | 1411.6911 | 1411.6894 | -1.2 |
| [b40+Na]3+ | 1444.7139 | 1444.7146 | 0.5 |
| [b41+Na]4+ | 1112.0583 | 1112.0583 | 0.0 |
| [y7+Na]+ | 636.3692 | 636.3681 | -1.7 |
| [y8+Na]+ | 767.4096 | 767.4103 | 0.9 |
| [y10+Na]+ | 937.5152 | 937.5162 | 1.1 |
| [y19+Na]2+ | 889.0045 | 889.0048 | 0.4 |
| [y31+Na]3+ | 1076.5840 | 1076.5836 | -0.4 |
| [y35+Na]3+ | 1221.9705 | 1221.9695 | -0.8 |
| [y39+Na]4+ | 1055.5435 | 1055.5456 | 2.0 |
| [y41+Na]4+ | 1105.5634 | 1105.5650 | 1.4 |
| [y42+Na]4+ | 1134.3202 | 1134.3187 | -1.3 |
| Average |  |  | -0.18 |
| St Dev |  |  | 1.15 |

**IR-ECD of [M+3H+Na]^4+^**

| **Fragment** | **Exact m/z** | **Observed m/z** | **Mass error (ppm)** |
| --- | --- | --- | --- |
| [c5]+ | 636.3100 | 636.3101 | 0.0 |
| [c6]+ | 773.3690 | 773.3690 | 0.0 |
| [c7]+ | 888.3959 | 888.3957 | -0.2 |
| [c8]+ | 975.4279 | 975.4280 | 0.1 |
| [c9]+ | 1032.4494 | 1032.4493 | -0.1 |
| [c10]+ | 1195.5127 | 1195.5130 | 0.3 |
| [c11]+ | 1324.5553 | 1324.5555 | 0.1 |
| [c12]+ | 1423.6237 | 1423.6236 | -0.1 |
| [c13]+ | 1560.6826 | 1560.6845 | 1.2 |
| [c14]+ | 1697.7416 | 1697.7414 | -0.1 |
| [c18]+ | 2166.0476 | 2166.0477 | 0.1 |
| [c22-H]2+ | 1330.1321 | 1330.1320 | 0.0 |
| [c23]2+ | 1388.1492 | 1388.1507 | 1.1 |
| [c24-H]2+ | 1437.1797 | 1437.1802 | 0.3 |
| [c25-H]2+ | 1465.6905 | 1465.6912 | 0.5 |
| [c26]2+ | 1509.7101 | 1509.7092 | -0.6 |
| [c27]2+ | 1566.7316 | 1566.7313 | -0.2 |
| [c28]2+ | 1630.7791 | 1630.7786 | -0.3 |
| [c29]2+ | 1659.2898 | 1659.2899 | 0.1 |
| [c30]2+ | 1694.8084 | 1694.8086 | 0.2 |
| [c31]2+ | 1751.3504 | 1751.3520 | 0.9 |
| [c32]2+ | 1807.8924 | 1807.8929 | 0.3 |
| [c33]2+ | 1836.4032 | 1836.4036 | 0.2 |
| [c34-H]2+ | 1892.4415 | 1892.4429 | 0.7 |
| [c35-H]2+ | 1957.9618 | 1957.9601 | -0.9 |
| [c36-H]2+ | 2007.4960 | 2007.4956 | -0.2 |
| [c38-H]2+ | 2064.5174 | 2064.5197 | 1.1 |
| [c9+Na]+ | 1054.4313 | 1054.4313 | 0.0 |
| [c10+Na]+ | 1217.4946 | 1217.4942 | -0.3 |
| [c11+Na]+ | 1346.5372 | 1346.5352 | -1.5 |
| [c36+Na]2+ | 2018.9906 | 2018.9919 | 0.7 |
| [c37+Na]2+ | 2047.5013 | 2047.5020 | 0.3 |
| [c38+Na]2+ | 2076.0121 | 2076.0130 | 0.5 |
| [c39+Na]2+ | 2125.5463 | 2125.5472 | 0.5 |
| [c38+Na-H]3+ | 1384.0080 | 1384.0087 | 0.5 |
| [c39+Na-H]3+ | 1417.0308 | 1417.0311 | 0.2 |
| [c40+Na]3+ | 1450.3894 | 1450.3891 | -0.2 |
| [c41+Na]3+ | 1488.0841 | 1488.0842 | 0.1 |
| [z7+Na]+ | 620.3510 | 620.3505 | -0.8 |
| [z8+Na]+ | 751.3915 | 751.3906 | -1.2 |
| [z9+Na]+ | 864.4756 | 864.4748 | -0.9 |
| [z10+Na]+ | 921.4970 | 921.4959 | -1.2 |
| [z11+Na]+ | 1034.5811 | 1034.5807 | -0.4 |
| [z12+Na]+ | 1147.6651 | 1147.6653 | 0.2 |
| [z13+Na]+ | 1218.7023 | 1218.7013 | -0.8 |
| [z14+Na]+ | 1275.7237 | 1275.7228 | -0.8 |
| [z15+Na]+ | 1403.8187 | 1403.8182 | -0.3 |
| [z16+Na]+ | 1517.8616 | 1517.8611 | -0.4 |
| [z17+Na+H]+ | 1605.9009 | 1605.9005 | -0.3 |
| [z18+Na+H]+ | 1662.9224 | 1662.9221 | -0.2 |
| [z20+Na+H]+ | 1877.0178 | 1877.0173 | -0.3 |
| [z30+Na]2+ | 1556.8291 | 1556.8303 | 0.8 |
| [z31+Na]2+ | 1606.3633 | 1606.3615 | -1.1 |
| [z32+Na]2+ | 1670.8846 | 1670.8851 | 0.3 |
| [z33+Na]2+ | 1752.4162 | 1752.4173 | 0.6 |
| [z34+Na]2+ | 1780.9270 | 1780.9261 | -0.5 |
| [z35+Na]2+ | 1824.4430 | 1824.4440 | 0.5 |
| [z36+Na]2+ | 1881.9565 | 1881.9572 | 0.4 |
| [z37+Na]2+ | 1950.4859 | 1950.4868 | 0.5 |
| [z38+Na]2+ | 2028.5365 | 2028.5391 | 1.3 |
| [z39+Na]2+ | 2102.0707 | 2102.0642 | -3.1 |
| [z40+Na+H]2+ | 2167.0956 | 2167.0992 | 1.6 |
| [z41+Na]2+ | 2202.1105 | 2202.1098 | -0.3 |
| [z39+Na]3+ | 1401.7162 | 1401.7130 | -2.3 |
| [z40+Na]3+ | 1444.7304 | 1444.7294 | -0.7 |
| [z41+Na]3+ | 1468.4094 | 1468.4097 | 0.1 |
| Average |  |  | -0.03 |
| St Dev |  |  | 0.81 |

**CID (30 V) of [M+2H+Ca]^4+^**

| **Fragment** | **Exact m/z** | **Observed m/z** | **Mass error (ppm)** |
| --- | --- | --- | --- |
| [b5]+ | 619.2835 | 619.2821 | -2.2 |
| [b6]+ | 756.3424 | 756.3419 | -0.7 |
| [b7]+ | 871.3694 | 871.3694 | 0.0 |
| [b11]+ | 1307.5288 | 1307.5281 | -0.5 |
| [b12]+ | 1406.5972 | 1406.5975 | 0.2 |
| [b13]+ | 1543.6561 | 1543.6558 | -0.2 |
| [b13]2+ | 772.3317 | 772.3305 | -1.5 |
| [b14]2+ | 840.8612 | 840.8603 | -1.0 |
| [b15]2+ | 904.8904 | 904.8899 | -0.6 |
| [b16]2+ | 968.9379 | 968.9385 | 0.6 |
| [b18]2+ | 1075.0142 | 1075.0153 | 1.1 |
| [b19]2+ | 1148.5484 | 1148.5524 | 3.5 |
| [b20]2+ | 1222.0826 | 1222.0837 | 0.9 |
| [b22]2+ | 1322.1224 | 1322.1217 | -0.6 |
| [b23]2+ | 1379.6359 | 1379.6365 | 0.4 |
| [b24]3+ | 953.1158 | 953.1161 | 0.3 |
| [b25]3+ | 972.1230 | 972.1240 | 1.1 |
| [b30]3+ | 1124.5325 | 1124.5375 | 4.4 |
| [b31]3+ | 1162.2272 | 1162.2253 | -1.6 |
| [b32]3+ | 1199.9219 | 1199.9217 | -0.2 |
| [b33]3+ | 1218.9290 | 1218.9291 | 0.1 |
| [b34]3+ | 1256.6237 | 1256.6233 | -0.3 |
| [b35]3+ | 1300.3039 | 1300.3030 | -0.7 |
| [b36]3+ | 1333.3267 | 1333.3266 | 0.0 |
| [b39]3+ | 1404.3638 | 1404.3675 | 2.6 |
| [b33]4+ | 914.4486 | 914.4483 | -0.3 |
| [b34]4+ | 942.7196 | 942.7218 | 2.3 |
| [b35]4+ | 975.4797 | 975.4798 | 0.1 |
| [b37]4+ | 1014.5022 | 1014.5007 | -1.5 |
| [b39+Ca]4+ | 1063.0114 | 1063.0127 | 1.2 |
| [b40+Ca]4+ | 1087.7785 | 1087.7779 | -0.5 |
| [b41+Ca]4+ | 1116.0495 | 1116.0496 | 0.0 |
| [y10+Ca]+ | 953.4802 | 953.4799 | -0.3 |
| [y19+Ca]2+ | 896.9870 | 896.9863 | -0.8 |
| [y23+Ca]2+ | 1128.0745 | 1128.0746 | 0.1 |
| [y24+Ca]2+ | 1201.6087 | 1201.6084 | -0.3 |
| [y25+Ca]2+ | 1251.1429 | 1251.1422 | -0.6 |
| [y26+Ca]2+ | 1307.6850 | 1307.6844 | -0.5 |
| [y27+Ca]2+ | 1371.7325 | 1371.7318 | -0.5 |
| [y28+Ca]2+ | 1435.7618 | 1435.7593 | -1.7 |
| [y29+Ca]2+ | 1504.2912 | 1504.2904 | -0.5 |
| [y29+Ca]3+ | 1003.1966 | 1003.1963 | -0.2 |
| [y30+Ca]3+ | 1048.8829 | 1048.8871 | 4.1 |
| [y31+Ca]3+ | 1081.9057 | 1081.9058 | 0.1 |
| [y35+Ca]3+ | 1227.2922 | 1227.2925 | 0.3 |
| [y39+Ca]4+ | 1059.5348 | 1059.5352 | 0.4 |
| [y41+Ca]4+ | 1109.5547 | 1109.5544 | -0.3 |
| Average |  |  | 0.12 |
| St Dev |  |  | 1.36 |

**IRMPD of [M+2H+Ca]^4+^**

| **Fragment** | **Exact m/z** | **Observed m/z** | **Mass error (ppm)** |
| --- | --- | --- | --- |
| [b6]+ | 756.3424 | 756.3424 | 0.0 |
| [b7]+ | 871.3694 | 871.3695 | 0.1 |
| [b11]+ | 1307.5288 | 1307.5265 | -1.8 |
| [b23]2+ | 1379.6359 | 1379.635 | -1.0 |
| [b41+Ca]4+. | 1116.0495 | 1116.051 | 1.1 |
| [y14+Ca]2+ | 654.3571 | 654.357 | -0.4 |
| [y15+Ca]2+ | 718.4046 | 718.404 | -0.3 |
| [y18+Ca]2+ | 847.4528 | 847.452 | -1.3 |
| [y19+Ca]2+ | 896.987 | 896.988 | 0.8 |
| [y31+Ca]3+ | 1081.9057 | 1081.907 | 0.9 |
| [y35+Ca]3+ | 1227.2922 | 1227.292 | 0.2 |
| [y39+Ca]4+ | 1059.5348 | 1059.536 | 0.9 |
| [y41+Ca]4+ | 1109.5547 | 1109.556 | 0.7 |
| Average |  |  | 0.01 |
| St Dev |  |  | 0.92 |

**IR-ECD of [M+2H+Ca]^4+^**

| **Fragment** | **Exact m/z** | **Observed m/z** | **Mass error (ppm)** |
| --- | --- | --- | --- |
| [c5]+ | 636.31004 | 636.31002 | 0.0 |
| [c6]+ | 773.36896 | 773.36893 | 0.0 |
| [c7]+ | 888.3959 | 888.3962 | 0.3 |
| [c10]+ | 1195.51272 | 1195.51324 | 0.4 |
| [c37+Ca]2+ | 2055.4838 | 2055.48282 | -0.5 |
| [c38+Ca]3+ | 1389.6655 | 1389.6657 | 0.1 |
| [c39+Ca]3+ | 1422.6883 | 1422.68615 | -1.5 |
| [c40+Ca]3+ | 1455.7111 | 1455.71072 | -0.3 |
| [c41+Ca]3+ | 1493.4058 | 1493.40449 | -0.9 |
| [z33+Ca]2+ | 1760.3988 | 1760.4001 | 0.8 |
| [z35+Ca]2+ | 1832.4255 | 1832.4282 | 1.5 |
| [z36+Ca]2+ | 1889.9390 | 1889.93915 | 0.1 |
| [z37+Ca]2+ | 1958.4684 | 1958.46702 | -0.7 |
| [z38+Ca]2+ | 2036.5190 | 2036.525 | 3.0 |
| Average |  |  | 0.16 |
| St Dev |  |  | 1.10 |

**CID (30 V) of [M+2H+Mg]^4+^**

| **Fragment** | **Exact m/z** | **Observed m/z** | **Mass error (ppm)** |
| --- | --- | --- | --- |
| [b5]+ | 619.2835 | 619.2852 | 2.8 |
| [b6]+ | 756.3424 | 756.3426 | 0.3 |
| [b7]+ | 871.3694 | 871.3692 | -0.1 |
| [b11]+ | 1307.5288 | 1307.5278 | -0.7 |
| [b12]+ | 1406.5972 | 1406.5965 | -0.5 |
| [b13]+ | 1543.6561 | 1543.6584 | 1.5 |
| [b13]2+ | 772.3317 | 772.3320 | 0.3 |
| [b14]2+ | 840.8612 | 840.8611 | -0.1 |
| [b15]2+ | 904.8904 | 904.8908 | 0.4 |
| [b16]2+ | 968.9379 | 968.9377 | -0.3 |
| [b18]2+ | 1075.0142 | 1075.0151 | 0.8 |
| [b20]2+ | 1222.0826 | 1222.0828 | 0.2 |
| [b22]2+ | 1322.1224 | 1322.1229 | 0.3 |
| [b23]2+ | 1379.6359 | 1379.6368 | 0.6 |
| [b24]3+ | 953.1158 | 953.1161 | 0.3 |
| [b25]3+ | 972.1230 | 972.1224 | -0.6 |
| [b28]3+ | 1081.8463 | 1081.8429 | -3.1 |
| [b30]3+ | 1124.5325 | 1124.5301 | -2.1 |
| [b31]3+ | 1162.2272 | 1162.2263 | -0.7 |
| [b32]3+ | 1199.9219 | 1199.9213 | -0.4 |
| [b33]4+ | 914.4486 | 914.4492 | 0.6 |
| [b38]4+ | 1028.7576 | 1028.7591 | 1.4 |
| [b38+Mg]4+ | 1034.2499 | 1034.2486 | -1.2 |
| [b39+Mg]4+ | 1059.0170 | 1059.0159 | -1.1 |
| [b40+Mg]4+ | 1083.7841 | 1083.7836 | -0.5 |
| [b41+Mg]4+ | 1112.0551 | 1112.0545 | -0.6 |
| [y11+Mg]+ | 1050.5867 | 1050.5854 | -1.3 |
| [y19+Mg]2+ | 888.9982 | 888.9974 | -0.9 |
| [y26+Mg]2+ | 1299.6962 | 1299.6944 | -1.4 |
| [y29+Mg]3+ | 997.8707 | 997.8694 | -1.4 |
| [y31+Mg]3+ | 1076.5798 | 1076.5781 | -1.6 |
| [y35+Mg]3+ | 1221.9663 | 1221.9656 | -0.5 |
| [y41+Mg]4+ | 1105.5603 | 1105.5614 | 1.0 |
| Average |  |  | -0.26 |
| St Dev |  |  | 1.13 |

**IRMPD of [M+2H+Mg]^4+^**

| **Fragment** | **Exact m/z** | **Observed m/z** | **Mass error (ppm)** |
| --- | --- | --- | --- |
| [b6]+ | 756.3424 | 756.3424 | 0.0 |
| [b7]+ | 871.3694 | 871.3685 | -1.0 |
| [b11]+ | 1307.5288 | 1307.5301 | 1.0 |
| [b41+Mg]4+ | 1112.0551 | 1112.0553 | 0.1 |
| [y19+Mg]2+ | 888.9982 | 888.9985 | 0.3 |
| [y31+Mg]3+ | 1076.5798 | 1076.5815 | 1.5 |
| [y35+Mg]3+ | 1221.9663 | 1221.9645 | -1.5 |
| [y41+Mg]4+ | 1105.5603 | 1105.5610 | 0.6 |
| Average |  |  | 0.13 |
| St Dev |  |  | 0.99 |

**IR-ECD of [M+2H+Mg]^4+^**

| **Fragment** | **Exact m/z** | **Observed m/z** | **Mass error (ppm)** |
| --- | --- | --- | --- |
| [c5]+ | 636.31004 | 636.30989 | -0.2 |
| [c6]+ | 773.36896 | 773.36962 | 0.9 |
| [c7]+ | 888.3959 | 888.3956 | -0.3 |
| [c8]+ | 975.42793 | 975.42764 | -0.3 |
| [c10]+ | 1195.5127 | 1195.51202 | -0.6 |
| [c37+Mg+H]3+ | 1365.6682 | 1365.6680 | -0.2 |
| [c39+Mg]3+ | 1417.3624 | 1417.36417 | 1.2 |
| [c40+Mg]3+ | 1450.3852 | 1450.3865 | 0.9 |
| [c41+Mg]3+ | 1488.0799 | 1488.07934 | -0.4 |
| [z31+Mg+H]3+ | 1606.8607 | 1606.86011 | -0.3 |
| [z35+Mg+H]3+ | 1824.9404 | 1824.93706 | -1.8 |
| [z36+Mg]3+ | 1881.9502 | 1881.95059 | 0.2 |
| [z37+Mg+H]3+ | 1950.9833 | 1950.98104 | -1.2 |
| [z39+Mg+H]3+ | 2102.5681 | 2102.56594 | -1.0 |
| Average |  |  | -0.22 |
| St Dev |  |  | 0.83 |

**CID (30 V) of [M+2H+Mn]^4+^**

| **Fragment** | **Exact m/z** | **Observed m/z** | **Mass error (ppm)** |
| --- | --- | --- | --- |
| [b5]+ | 619.2835 | 619.2836 | 0.2 |
| [b6]+ | 756.3424 | 756.3427 | 0.3 |
| [b7]+ | 871.3694 | 871.3693 | 0.0 |
| [b11]+ | 1307.5288 | 1307.5288 | 0.0 |
| [b12]+ | 1406.5972 | 1406.5970 | -0.1 |
| [b11]2+ | 654.2680 | 654.2685 | 0.7 |
| [b13]2+ | 772.3317 | 772.3317 | 0.0 |
| [b14]2+ | 840.8612 | 840.8612 | 0.0 |
| [b15]2+ | 904.8904 | 904.8905 | 0.1 |
| [b16]2+ | 968.9379 | 968.9374 | -0.5 |
| [b18]2+ | 1075.0142 | 1075.0138 | -0.4 |
| [b20]2+ | 1222.0826 | 1222.0805 | -1.7 |
| [b22]2+ | 1322.1224 | 1322.1210 | -1.1 |
| [b23]2+ | 1379.6359 | 1379.6359 | 0.0 |
| [b24]3+ | 953.1158 | 953.1159 | 0.0 |
| [b25]3+ | 972.1230 | 972.1230 | 0.0 |
| [b27]3+ | 1039.1480 | 1039.1477 | -0.2 |
| [b30]3+ | 1124.5325 | 1124.5317 | -0.7 |
| [b31]3+ | 1162.2272 | 1162.2256 | -1.3 |
| [b32]3+ | 1199.9219 | 1199.9204 | -1.2 |
| [b33]3+ | 1218.9290 | 1218.9292 | 0.1 |
| [b34]3+ | 1256.6237 | 1256.6251 | 1.1 |
| [b36]3+ | 1333.3267 | 1333.3245 | -1.6 |
| [b33]4+ | 914.4486 | 914.4475 | -1.1 |
| [b34]4+ | 942.7196 | 942.7194 | -0.2 |
| [b38]4+ | 1028.7576 | 1028.7572 | -0.4 |
| [b39+Mn(II)]3+ | 1422.0046 | 1422.0055 | 0.7 |
| [b35+Mn(II)]4+ | 988.7103 | 988.7090 | -1.3 |
| [b36+Mn(II)]4+ | 1013.4774 | 1013.4767 | -0.7 |
| [b38+Mn(II)]4+ | 1041.9882 | 1041.9881 | 0.0 |
| [b39+Mn(II)]4+ | 1066.7553 | 1066.7554 | 0.1 |
| [b40+Mn(II)]4+ | 1091.5224 | 1091.5223 | 0.0 |
| [b41+Mn(II)]4+ | 1119.7934 | 1119.7934 | 0.0 |
| [y10+Mn(II)]+ | 968.4556 | 968.4556 | -0.1 |
| [y11+Mn(II)]+ | 1081.5397 | 1081.5381 | -1.5 |
| [y18+Mn(II)]2+ | 854.9405 | 854.9409 | 0.5 |
| [y19+Mn(II)]2+ | 904.4747 | 904.4749 | 0.2 |
| [y31+Mn(II)]3+ | 1086.8975 | 1086.8986 | 1.1 |
| [y35+Mn(II)]3+ | 1232.2840 | 1232.2848 | 0.7 |
| [y41+Mn(II)]4+ | 1113.2986 | 1113.2998 | 1.1 |
| Average |  |  | 0.18 |
| St Dev |  |  | 0.73 |

**IRMPD of [M+2H+Mn]^4+^**

| **Fragment** | **Exact m/z** | **Observed m/z** | **Mass error (ppm)** |
| --- | --- | --- | --- |
| [b6]+ | 756.3424 | 756.3425 | 0.1 |
| [b7]+ | 871.3694 | 871.3691 | -0.3 |
| [b23]2+ | 1379.6359 | 1379.6356 | -0.2 |
| [b41+Mn(II)]4+ | 1119.7934 | 1119.7939 | 0.4 |
| [y19+Mn(II)]2+ | 904.4747 | 904.4740 | -0.8 |
| [y35+Mn(II)]3+ | 1232.2840 | 1232.2830 | -0.8 |
| [y39+Mn(II)]4+ | 1063.2786 | 1063.2796 | 0.9 |
| [y41+Mn(II)]4+ | 1113.2986 | 1113.2983 | -0.3 |
| Average |  |  | -0.13 |
| St Dev |  |  | 0.58 |

**IR-ECD of [M+2H+Mn]^4+^**

| **Fragment** | **Exact m/z** | **Observed m/z** | **Mass error (ppm)** |
| --- | --- | --- | --- |
| [c6]+ | 636.3100 | 636.3101 | 0.1 |
| [c7]+ | 773.3690 | 773.3688 | -0.2 |
| [c8]+ | 888.3959 | 888.3957 | -0.2 |
| [c38+Mn(II)]3+ | 1394.6573 | 1394.6574 | 0.0 |
| [c39+Mn(II)]3+ | 1427.6801 | 1427.6772 | -2.1 |
| [c40+Mn(II)]3+ | 1460.7029 | 1460.7058 | 2.0 |
| [z33+Mn(II)]2+ | 1767.8865 | 1767.8864 | -0.1 |
| [z38+Mn(II)]2+ | 2044.0067 | 2044.0084 | 0.9 |
| Average |  |  | 0.05 |
| St Dev |  |  | 1.15 |

**CID (30 V) of [M+2H+Ni]^4+^**

| **Fragment** | **Exact m/z** | **Observed m/z** | **Mass error (ppm)** |
| --- | --- | --- | --- |
| [b6]+ | 756.3424 | 756.3426 | 0.3 |
| [b7]+ | 871.3694 | 871.3690 | -0.4 |
| [b12]+ | 1406.5972 | 1406.5981 | 0.7 |
| [b13]+ | 1543.6561 | 1543.6550 | -0.7 |
| [b12]2+ | 703.8022 | 703.8026 | 0.5 |
| [b13]2+ | 772.3317 | 772.3310 | -0.9 |
| [b14]2+ | 840.8612 | 840.8608 | -0.4 |
| [b15]2+ | 904.8904 | 904.8911 | 0.7 |
| [b16]2+ | 968.9379 | 968.9376 | -0.3 |
| [b18]2+ | 1075.0142 | 1075.0146 | 0.4 |
| [b23]2+ | 1379.6359 | 1379.6370 | 0.8 |
| [b24]2+ | 1429.1701 | 1429.1690 | -0.8 |
| [b27]2+ | 1558.2183 | 1558.2172 | -0.7 |
| [b27]3+ | 1039.1480 | 1039.1481 | 0.2 |
| [b35]3+ | 1300.3039 | 1300.3046 | 0.6 |
| [y7]+ | 614.3872 | 614.3872 | -0.1 |
| [y8]+ | 745.4277 | 745.4282 | 0.6 |
| [y10]+ | 915.5332 | 915.5328 | -0.5 |
| [b6+Ni(II)]+ | 812.2621 | 812.2632 | 1.3 |
| [b7+Ni(II)]+ | 927.2890 | 927.2892 | 0.2 |
| [b12+Ni(II)]+ | 1462.5169 | 1462.5152 | -1.1 |
| [b13+Ni(II)]+ | 1599.5758 | 1599.5754 | -0.2 |
| [b14+Ni(II)]+ | 1736.6347 | 1736.6346 | 0.0 |
| [b13+Ni(II)]2+ | 800.2915 | 800.2922 | 0.9 |
| [b14+Ni(II)]2+ | 868.8210 | 868.8208 | -0.3 |
| [b15+Ni(II)]2+ | 932.8503 | 932.8505 | 0.2 |
| [b18+Ni(II)]2+ | 1102.9740 | 1102.9741 | 0.1 |
| [b20+Ni(II)]2+ | 1250.0424 | 1250.0434 | 0.8 |
| [b22+Ni(II)]2+ | 1350.0823 | 1350.0838 | 1.1 |
| [b23+Ni(II)]2+ | 1407.5957 | 1407.5972 | 1.0 |
| [b25+Ni(II)]2+ | 1485.6407 | 1485.6385 | -1.5 |
| [b27+Ni(II)]2+ | 1586.1782 | 1586.1810 | 1.8 |
| [b20+Ni(II)]3+ | 833.6974 | 833.6974 | 0.0 |
| [b21+Ni(II)]3+ | 857.3764 | 857.3764 | -0.1 |
| [b22+Ni(II)]3+ | 900.3906 | 900.3896 | -1.1 |
| [b23+Ni(II)]3+ | 938.7329 | 938.7325 | -0.4 |
| [b24+Ni(II)]3+ | 971.7557 | 971.7556 | -0.2 |
| [b27+Ni(II)]3+ | 1057.7879 | 1057.7883 | 0.4 |
| [b30+Ni(II)]3+ | 1143.1724 | 1143.1724 | 0.0 |
| [b31+Ni(II)]3+ | 1180.8671 | 1180.8674 | 0.3 |
| [b32+Ni(II)]3+ | 1218.5618 | 1218.5624 | 0.5 |
| [b33+Ni(II)]3+ | 1237.5689 | 1237.5700 | 0.8 |
| [b34+Ni(II)]3+ | 1275.2636 | 1275.2647 | 0.8 |
| [b35+Ni(II)]3+ | 1318.9438 | 1318.9449 | 0.8 |
| [b36+Ni(II)]3+ | 1351.9666 | 1351.9673 | 0.6 |
| [b39+Ni(II)]3+ | 1423.0037 | 1423.0049 | 0.8 |
| [b40+Ni(II)]3+ | 1456.0265 | 1456.0265 | 0.0 |
| [b31+Ni(II)]4+ | 885.9021 | 885.9022 | 0.1 |
| [b32+Ni(II)]4+ | 914.1731 | 914.1737 | 0.6 |
| [b33+Ni(II)]4+ | 928.4285 | 928.4285 | 0.0 |
| [b34+Ni(II)]4+ | 956.6995 | 956.6999 | 0.4 |
| [b35+Ni(II)]4+ | 989.4596 | 989.4598 | 0.2 |
| [b37+Ni(II)]4+ | 1028.4821 | 1028.4826 | 0.5 |
| [b38+Ni(II)]4+ | 1042.7375 | 1042.7373 | -0.2 |
| [b40+Ni(II)]4+ | 1092.2717 | 1092.2722 | 0.5 |
| [b41+Ni(II)]4+ | 1120.5427 | 1120.5433 | 0.6 |
| Average |  |  | 0.18 |
| St Dev |  |  | 0.64 |

**IRMPD of [M+2H+Ni]^4+^**

| **Fragment** | **Exact m/z** | **Observed m/z** | **Mass error (ppm)** |
| --- | --- | --- | --- |
| [b5]+ | 619.2835 | 619.2836 | 0.1 |
| [b6]+ | 756.3424 | 756.3424 | 0.0 |
| [b7]+ | 871.3694 | 871.3692 | -0.2 |
| [b11]+ | 1307.5288 | 1307.5289 | 0.1 |
| [b12]+ | 1406.5972 | 1406.5971 | -0.1 |
| [b11]2+ | 654.2680 | 654.2684 | 0.6 |
| [b12]2+ | 703.8022 | 703.8022 | -0.1 |
| [b14]2+ | 840.8612 | 840.8614 | 0.2 |
| [y6]+ | 515.3188 | 515.3195 | 1.4 |
| [y7]+ | 614.3872 | 614.3876 | 0.7 |
| [y8]+ | 745.4277 | 745.4274 | -0.5 |
| [y10]+ | 915.5332 | 915.5330 | -0.3 |
| [y11]+ | 1028.6173 | 1028.6170 | -0.3 |
| [y19]2+ | 878.0135 | 878.0134 | -0.1 |
| [b6+Ni(II)]+ | 812.2621 | 812.2628 | 0.8 |
| [b14+Ni(II)]2+ | 868.8210 | 868.8205 | -0.6 |
| [b22+Ni(II)]2+ | 1350.0823 | 1350.0843 | 1.5 |
| [b23+Ni(II)]2+ | 1407.5957 | 1407.6010 | 3.7 |
| [b23+Ni(II)]3+ | 938.7329 | 938.7326 | -0.3 |
| [b28+Ni(II)]3+ | 1100.4862 | 1100.4852 | -0.9 |
| [b29+Ni(II)]3+ | 1119.4933 | 1119.4924 | -0.8 |
| [b31+Ni(II)]3+ | 1180.8671 | 1180.8649 | -1.9 |
| [b32+Ni(II)]3+ | 1218.5618 | 1218.5605 | -1.0 |
| [b34+Ni(II)]3+ | 1275.2636 | 1275.2622 | -1.1 |
| [b36+Ni(II)]3+ | 1351.9666 | 1351.9648 | -1.3 |
| [b39+Ni(II)]3+ | 1423.0037 | 1423.0039 | 0.1 |
| [b40+Ni(II)]3+ | 1456.0265 | 1456.0271 | 0.5 |
| [b38+Ni(II)]4+ | 1042.7375 | 1042.7379 | 0.4 |
| [b39+Ni(II)]4+ | 1067.5046 | 1067.5038 | -0.8 |
| [b40+Ni(II)]4+ | 1092.2717 | 1092.2713 | -0.4 |
| [b41+Ni(II)]4+ | 1120.5427 | 1120.5424 | -0.3 |
| [y41+Ni(II)]4+ | 1114.0479 | 1114.0462 | -1.6 |
| Average |  |  | -0.08 |
| St Dev |  |  | 1.03 |

**IR-ECD of [M+2H+Ni]^4+^**

| **Fragment** | **Exact m/z** | **Observed m/z** | **Mass error (ppm)** |
| --- | --- | --- | --- |
| [c5]+ | 636.3100 | 636.3101 | 0.1 |
| [c6]+ | 773.3690 | 773.3689 | -0.1 |
| [c7]+ | 888.3959 | 888.3959 | 0.0 |
| [c8]+ | 975.4279 | 975.4281 | 0.2 |
| [c9]+ | 1032.4494 | 1032.4488 | -0.5 |
| [c10]+ | 1195.5127 | 1195.5124 | -0.3 |
| [c11]+ | 1324.5553 | 1324.5565 | 0.9 |
| [c12]+ | 1423.6237 | 1423.6253 | 1.1 |
| [z15+H]+ | 1382.8441 | 1382.8443 | 0.2 |
| [z16+H]+ | 1496.8870 | 1496.8866 | -0.3 |
| [z17+H]+ | 1583.9190 | 1583.9207 | 1.1 |
| [z18+H]+ | 1640.9405 | 1640.9444 | 2.4 |
| [z19+H]+ | 1740.0089 | 1740.0024 | -3.7 |
| [z20+H]+ | 1855.0358 | 1855.0357 | -0.1 |
| [z23+H]+ | 2202.1840 | 2202.1857 | 0.8 |
| [z24+H]+ | 2349.2524 | 2349.2606 | 3.5 |
| [z28]2+ | 1408.7792 | 1408.7803 | 0.8 |
| [z30+H]2+ | 1546.3418 | 1546.3449 | 2.0 |
| [z31+H]2+ | 1595.8760 | 1595.8810 | 3.2 |
| [c14+Ni(I)-H]+ | 1753.6618 | 1753.6655 | 2.1 |
| [c22+Ni(I)-H]2+ | 1358.5958 | 1358.5904 | -4.0 |
| [c25+Ni(I)]2+ | 1494.6579 | 1494.6539 | -2.6 |
| [c26+Ni(I)]2+ | 1538.1739 | 1538.1701 | -2.5 |
| [c33+Ni(I)]2+ | 1864.8669 | 1864.8635 | -1.8 |
| [z30+Ni(I)]2+ | 1574.3019 | 1574.3000 | -1.2 |
| [z32+Ni(I)]2+ | 1688.3574 | 1688.3569 | -0.3 |
| [z33+Ni(I)]2+ | 1769.8890 | 1769.8850 | -2.3 |
| [z34+Ni(I)+H]2+ | 1798.9034 | 1798.8969 | -3.6 |
| [z41+Ni(I)+H]2+ | 2220.0870 | 2220.0840 | -1.3 |
| [z41+Ni(I)]3+ | 1480.0580 | 1480.0561 | -1.2 |
| Average |  |  | -0.25 |
| St Dev |  |  | 1.94 |

**CID (30 V) of [M+2H+Co]^4+^**

| **Fragment** | **Exact m/z** | **Observed m/z** | **Mass error (ppm)** |
| --- | --- | --- | --- |
| [b6]+ | 756.34241 | 756.34251 | 0.1 |
| [b7]+ | 871.36935 | 871.3691 | -0.3 |
| [b11]+ | 1307.52876 | 1307.52924 | 0.4 |
| [b12]+ | 1406.59718 | 1406.59772 | 0.4 |
| [b13]+ | 1543.6561 | 1543.6552 | -0.6 |
| [b23]2+ | 1379.6359 | 1379.6365 | 0.5 |
| [b13+Co(II)]2+ | 800.7905 | 800.7905 | 0.0 |
| [b14+Co(II)]2+ | 869.3199 | 869.3194 | -0.6 |
| [b31+Co(II)]3+ | 1181.1997 | 1181.1988 | -0.8 |
| [b33+Co(II)]3+ | 1237.9015 | 1237.9012 | -0.3 |
| [b34+Co(II)]3+ | 1275.5962 | 1275.5960 | -0.2 |
| [b35+Co(II)]3+ | 1319.2764 | 1319.2764 | 0.0 |
| [b38+Co(II)]3+ | 1390.3135 | 1390.3132 | -0.2 |
| [b39+Co(II)]3+ | 1423.3363 | 1423.3365 | 0.2 |
| [b40+Co(II)]3+ | 1456.3591 | 1456.3592 | 0.1 |
| [b33+Co(II)]4+ | 928.6780 | 928.6779 | -0.1 |
| [b34+Co(II)]4+ | 956.9490 | 956.9488 | -0.3 |
| [b35+Co(II)]4+ | 989.7091 | 989.7089 | -0.2 |
| [b37+Co(II)]4+ | 1028.7316 | 1028.7314 | -0.2 |
| [b38+Co(II)]4+ | 1042.9869 | 1042.9866 | -0.3 |
| [b39+Co(II)]4+ | 1067.7541 | 1067.7538 | -0.2 |
| [b40+Co(II)]4+ | 1092.5212 | 1092.5208 | -0.3 |
| Average |  |  | -0.13 |
| St Dev |  |  | 0.33 |

**IRMPD of [M+2H+Co]^4+^**

| **Fragment** | **Exact m/z** | **Observed m/z** | **Mass error (ppm)** |
| --- | --- | --- | --- |
| [b5]+ | 619.28349 | 619.2835 | 0.0 |
| [b6]+ | 756.34241 | 756.34241 | 0.0 |
| [b7]+ | 871.36935 | 871.36941 | 0.1 |
| [b11]+ | 1307.52876 | 1307.53036 | 1.2 |
| [b23]2+ | 1379.6359 | 1379.63565 | -0.2 |
| [b14+Co(II)]2+ | 869.3199 | 869.32003 | 0.1 |
| [b23+Co(II)]2+ | 1408.0947 | 1408.0942 | -0.3 |
| [b31+Co(II)]3+ | 1181.1997 | 1181.1993 | -0.3 |
| [b32+Co(II)]3+ | 1218.8944 | 1218.89346 | -0.8 |
| [b34+Co(II)]3+ | 1275.5962 | 1275.59651 | 0.2 |
| [b35+Co(II)]3+ | 1319.2764 | 1319.27462 | -1.3 |
| [b36+Co(II)]3+ | 1352.2992 | 1352.2977 | -1.1 |
| [b39+Co(II)]3+ | 1423.3363 | 1423.3358 | -0.4 |
| [b40+Co(II)]3+ | 1456.3591 | 1456.35875 | -0.2 |
| [b38+Co(II)]4+ | 1042.9869 | 1042.98684 | -0.1 |
| [b39+Co(II)]4+ | 1067.7541 | 1067.75423 | 0.2 |
| [b40+Co(II)]4+ | 1092.5212 | 1092.52121 | 0.1 |
| [b41+Co(II)]4+ | 1120.7922 | 1120.79192 | -0.2 |
| Average |  |  | -0.17 |
| St Dev |  |  | 0.55 |

**IR-ECD of [M+2H+Co]^4+^**

| **Fragment** | **Exact m/z** | **Observed m/z** | **Mass error (ppm)** |
| --- | --- | --- | --- |
| [c7]+ | 888.3959 | 888.39589 | 0.0 |
| [c25+Co(II)-H]2+ | 1494.149232 | 1494.1464 | -1.9 |
| [c38+Co(II)]3+ | 1395.989012 | 1395.98877 | -0.2 |
| [c39+Co(II)]3+ | 1429.011818 | 1429.01158 | -0.2 |
| [c40+Co(II)]3+ | 1462.0346 | 1462.03213 | -1.7 |
| [c41+Co(II)]3+ | 1499.7293 | 1499.7295 | 0.1 |
| [z40+Co(II)]3+ | 1456.3756 | 1456.3791 | 2.4 |
| Average |  |  | 0.21 |
| St Dev |  |  | 1.42 |

**CID (30 V) of [M+4H]^4+^**

| **Fragment** | **Exact m/z** | **Observed m/z** | **Mass error (ppm)** |
| --- | --- | --- | --- |
| [b5]+ | 619.2835 | 619.2840 | 0.8 |
| [b6]+ | 756.3424 | 756.3425 | 0.1 |
| [b7]+ | 871.3694 | 871.3692 | -0.2 |
| [b10]+ | 1178.4862 | 1178.4843 | -1.6 |
| [b11]+ | 1307.5288 | 1307.5286 | -0.1 |
| [b12]+ | 1406.5972 | 1406.5984 | 0.8 |
| [b13]+ | 1543.6561 | 1543.6561 | 0.0 |
| [b14]+ | 1680.7150 | 1680.7180 | 1.8 |
| [b11]2+ | 654.2680 | 654.2683 | 0.4 |
| [b12]2+ | 703.8022 | 703.8021 | -0.2 |
| [b13]2+ | 772.3317 | 772.3318 | 0.1 |
| [b14]2+ | 840.8612 | 840.8604 | -0.9 |
| [b15]2+ | 904.8904 | 904.8905 | 0.1 |
| [b16]2+ | 968.9379 | 968.9380 | 0.1 |
| [b18]2+ | 1075.0142 | 1075.0144 | 0.3 |
| [b19]2+ | 1148.5484 | 1148.5484 | 0.0 |
| [b20]2+ | 1222.0826 | 1222.0824 | -0.2 |
| [b22]2+ | 1322.1224 | 1322.1222 | -0.2 |
| [b23]2+ | 1379.6359 | 1379.6356 | -0.2 |
| [b24]2+ | 1429.1701 | 1429.1698 | -0.2 |
| [b25]2+ | 1457.6808 | 1457.6804 | -0.3 |
| [b26]2+ | 1501.1969 | 1501.2001 | 2.1 |
| [b27]2+ | 1558.2183 | 1558.2177 | -0.4 |
| [b18]3+ | 717.0119 | 717.0120 | 0.2 |
| [b19]3+ | 766.0347 | 766.0347 | 0.0 |
| [b20]3+ | 815.0575 | 815.0577 | 0.3 |
| [b21]3+ | 838.7365 | 838.7360 | -0.6 |
| [b22]3+ | 881.7507 | 881.7495 | -1.4 |
| [b23]3+ | 920.0930 | 920.0929 | -0.2 |
| [b24]3+ | 953.1158 | 953.1158 | -0.1 |
| [b25]3+ | 972.1230 | 972.1229 | -0.1 |
| [b26]3+ | 1001.1337 | 1001.1320 | -1.7 |
| [b27]3+ | 1039.1480 | 1039.1479 | -0.1 |
| [b28]3+ | 1081.8463 | 1081.8461 | -0.2 |
| [b29]3+ | 1100.8535 | 1100.8523 | -1.1 |
| [b30]3+ | 1124.5325 | 1124.5322 | -0.2 |
| [b31]3+ | 1162.2272 | 1162.2272 | 0.1 |
| [b32]3+ | 1199.9219 | 1199.9222 | 0.3 |
| [b33]3+ | 1218.9290 | 1218.9293 | 0.2 |
| [b34]3+ | 1256.6237 | 1256.6239 | 0.2 |
| [b35]3+ | 1300.3039 | 1300.3039 | 0.1 |
| [b36]3+ | 1333.3267 | 1333.3272 | 0.4 |
| [b37]3+ | 1352.3338 | 1352.3336 | -0.2 |
| [b38]3+ | 1371.3410 | 1371.3408 | -0.1 |
| [b39]3+ | 1404.3638 | 1404.3634 | -0.3 |
| [b40]3+ | 1437.3866 | 1437.3861 | -0.3 |
| [b30]4+ | 843.6512 | 843.6508 | -0.5 |
| [b31]4+ | 871.9222 | 871.9212 | -1.2 |
| [b32]4+ | 900.1932 | 900.1935 | 0.3 |
| [b33]4+ | 914.4486 | 914.4487 | 0.1 |
| [b34]4+ | 942.7196 | 942.7197 | 0.1 |
| [b35]4+ | 975.4797 | 975.4796 | -0.1 |
| [b36]4+ | 1000.2468 | 1000.2477 | 0.8 |
| [b37]4+ | 1014.5022 | 1014.5020 | -0.2 |
| [b38]4+ | 1028.7576 | 1028.7575 | -0.1 |
| [b39]4+ | 1053.5247 | 1053.5246 | -0.1 |
| [b40]4+ | 1078.2918 | 1078.2913 | -0.5 |
| [y6]+ | 515.3188 | 515.3194 | 1.1 |
| [y7]+ | 614.3872 | 614.3875 | 0.4 |
| [y8]+ | 745.4277 | 745.4283 | 0.8 |
| [y9]+ | 858.5118 | 858.5116 | -0.2 |
| [y10]+ | 915.5332 | 915.5320 | -1.4 |
| [y11]+ | 1028.6173 | 1028.6167 | -0.6 |
| Average |  |  | -0.06 |
| St Dev |  |  | 0.66 |

**IRMPD of [M+4H]^4+^**

| **Fragment** | **Exact m/z** | **Observed m/z** | **Mass error (ppm)** |
| --- | --- | --- | --- |
| [b6]+ | 756.3424 | 756.3425 | 0.1 |
| [b7]+ | 871.3694 | 871.3693 | 0.0 |
| [b23]2+ | 1379.6359 | 1379.6344 | -1.1 |
| [b31]3+ | 1162.2272 | 1162.2263 | -0.7 |
| [b32]3+ | 1199.9219 | 1199.9199 | -1.6 |
| [b34]3+ | 1256.6237 | 1256.6246 | 0.7 |
| [b36]3+ | 1333.3267 | 1333.3240 | -2.0 |
| [b39]3+ | 1404.3638 | 1404.3655 | 1.2 |
| [b39]4+ | 1053.5247 | 1053.5250 | 0.4 |
| [b40]4+ | 1078.2918 | 1078.2914 | -0.3 |
| [b41]4+ | 1106.5628 | 1106.5628 | 0.0 |
| Average |  |  | -0.32 |
| St Dev |  |  | 0.98 |

**IR-ECD of [M+4H]^4+^**

| **Fragment** | **Exact m/z** | **Observed m/z** | **Mass error (ppm)** |
| --- | --- | --- | --- |
| [c5]+ | 636.3100 | 636.3100 | -0.1 |
| [c6]+ | 773.3690 | 773.3694 | 0.5 |
| [c7]+ | 888.3959 | 888.3966 | 0.8 |
| [c8-H]+ | 974.4246 | 974.4229 | -1.8 |
| [c9]+ | 1032.4494 | 1032.4496 | 0.2 |
| [c10]+ | 1195.5127 | 1195.5124 | -0.3 |
| [c11]+ | 1324.5553 | 1324.5551 | -0.2 |
| [c12]+ | 1423.6237 | 1423.6225 | -0.9 |
| [c13]+ | 1560.6826 | 1560.6813 | -0.9 |
| [c34]2+ | 1892.9452 | 1892.9412 | -2.1 |
| [c35-H]2+ | 1957.9638 | 1957.9601 | -1.9 |
| [c36]2+ | 2007.9996 | 2007.9946 | -2.5 |
| [c37]2+ | 2036.5104 | 2036.5071 | -1.6 |
| [c39]2+ | 2114.5553 | 2114.5504 | -2.3 |
| [c39-H]3+ | 1409.7049 | 1409.7042 | -0.5 |
| [c40]3+ | 1443.0621 | 1443.0621 | 0.0 |
| [z17+H]+ | 1583.9145 | 1583.9160 | 1.0 |
| [z29+H]2+ | 1477.8100 | 1477.8101 | 0.1 |
| [z30+H]2+ | 1546.3395 | 1546.3405 | 0.7 |
| [z31]2+ | 1595.3720 | 1595.3712 | -0.5 |
| [z32]2+ | 1659.8933 | 1659.8934 | 0.0 |
| [z33]2+ | 1741.4250 | 1741.4239 | -0.6 |
| [z34+H]2+ | 1770.4374 | 1770.4359 | -0.8 |
| [z35]2+ | 1813.4517 | 1813.4500 | -0.9 |
| [z36]2+ | 1870.9652 | 1870.9634 | -1.0 |
| [z37]2+ | 1939.4946 | 1939.4936 | -0.6 |
| [z38+H]2+ | 2018.0469 | 2018.0479 | 0.5 |
| [z39+H]2+ | 2091.5811 | 2091.5800 | -0.5 |
| [z42+H]2+ | 2249.1344 | 2249.1319 | -1.1 |
| [z40]3+ | 1437.4029 | 1437.4033 | 0.3 |
| Average |  |  | -0.57 |
| St Dev |  |  | 0.93 |

**CID (30 V) of [M+H+Co(NH_3_)_6_]^4+^**

| **Fragment** | **Exact m/z** | **Observed m/z** | **Mass error (ppm)** |
| --- | --- | --- | --- |
| [b7]+ | 871.3694 | 871.3693 | 0.0 |
| [b11]+ | 1307.5288 | 1307.5290 | 0.2 |
| [b23]2+ | 1379.6359 | 1379.6357 | -0.1 |
| [b24]3+ | 953.1159 | 953.1172 | 1.4 |
| [a6]+ | 728.3475 | 728.3475 | -0.1 |
| [a20]2+ | 1208.0851 | 1208.0846 | -0.4 |
| [a24]2+ | 1415.1727 | 1415.1718 | -0.6 |
| [c7]+ | 888.3959 | 888.3952 | -0.8 |
| [c25]2+ | 1466.1941 | 1466.1931 | -0.7 |
| [b14+Co(II)]2+ | 869.3199 | 869.3203 | 0.4 |
| [b24+Co(II)]3+ | 972.0884 | 972.0884 | 0.0 |
| [b32+Co(II)]3+ | 1218.8944 | 1218.8948 | 0.3 |
| [b34+Co(II)]3+ | 1275.5962 | 1275.5952 | -0.8 |
| [b35+Co(II)]3+ | 1319.6044 | 1319.6027 | -1.3 |
| [b39+Co(II)]3+ | 1423.6643 | 1423.6652 | 0.6 |
| [b40+Co(II)]3+ | 1456.3591 | 1456.3574 | -1.2 |
| [b34+Co(II)]4+ | 956.9490 | 956.9489 | -0.1 |
| [b35+Co(II)]4+ | 989.7091 | 989.7099 | 0.7 |
| [b36+Co(II)]4+ | 1014.4762 | 1014.4751 | -1.1 |
| [b38+Co(II)]4+ | 1042.9870 | 1042.9873 | 0.3 |
| [b39+Co(II)]4+ | 1067.7541 | 1067.7537 | -0.4 |
| [b41+Co(II)]4+ | 1121.0382 | 1121.0386 | 0.3 |
| [a13+Co(II)+H]2+ | 787.2967 | 787.2968 | 0.1 |
| [a39+Co(II)]4+ | 1061.0013 | 1061.0025 | 1.1 |
| [c25+Co(II)]3+ | 996.7710 | 996.7710 | -0.1 |
| Average |  |  | -0.09 |
| St Dev |  |  | 0.68 |

**IRMPD of [M+Fe^3+^+NTA^3-^+4H]^4+^**

| **Fragment** | **Exact m/z** | **Observed m/z** | **Mass error (ppm)** |
| --- | --- | --- | --- |
| [b5]+ | 619.2835 | 619.2847 | 1.9 |
| [b6]+ | 756.3424 | 756.3424 | 0.0 |
| [b7]+ | 871.3694 | 871.3693 | -0.1 |
| [b12]+ | 1406.5972 | 1406.5968 | -0.3 |
| [y10]+ | 915.5332 | 915.5330 | -0.3 |
| [y19]2+ | 878.0135 | 878.0134 | -0.1 |
| [b25+Fe(III)]3+ | 989.7601 | 989.7591 | -1.1 |
| [b31+Fe(III)]3+ | 1179.8643 | 1179.8625 | -1.5 |
| [b32+Fe(III)]3+ | 1217.5590 | 1217.5617 | 2.2 |
| [b36+Fe(III)]3+ | 1350.9638 | 1350.9615 | -1.7 |
| [b40+Fe(III)]3+ | 1455.0237 | 1455.0247 | 0.7 |
| [b40+Fe(III)]4+ | 1091.5196 | 1091.5195 | -0.1 |
| [b41+Fe(III)]4+ | 1119.7906 | 1119.7918 | 1.0 |
| [y35+Fe(III)]3+ | 1232.2803 | 1232.2814 | 0.9 |
| [y41+Fe(III)]4+ | 1113.2958 | 1113.2978 | 1.7 |
| Average |  |  | 0.21 |
| St Dev |  |  | 1.18 |

**CID (25 V) of [M+H+Co(NH_3_)_6_]^4+^**

| **Fragment** | **Exact m/z** | **Observed m/z** | **Mass error (ppm)** |
| --- | --- | --- | --- |
| [b7]+ | 871.3694 | 871.3689 | -0.6 |
| [b23]2+ | 1379.6359 | 1379.6362 | 0.2 |
| [b26]2+ | 1501.1969 | 1501.1995 | 1.8 |
| [b27]2+ | 1558.7103 | 1558.7118 | 1.0 |
| [b24]3+ | 953.1159 | 953.1144 | -1.6 |
| [b31]3+ | 1162.2272 | 1162.2262 | -0.9 |
| [b32]3+ | 1199.9219 | 1199.9211 | -0.7 |
| [b34+H]3+ | 1256.9595 | 1256.9582 | -1.0 |
| [b35+H]3+ | 1300.6396 | 1300.6421 | 1.9 |
| [b36+H]3+ | 1333.6625 | 1333.6639 | 1.1 |
| [b39+H]3+ | 1404.6996 | 1404.7014 | 1.3 |
| [b35]4+ | 975.7257 | 975.7266 | 0.9 |
| [b38]4+ | 1028.7576 | 1028.7564 | -1.1 |
| [b39]4+ | 1053.5247 | 1053.5240 | -0.7 |
| [b40]4+ | 1078.2918 | 1078.2912 | -0.6 |
| [a6]+ | 728.3475 | 728.3475 | 0.0 |
| [a7]+ | 843.3744 | 843.3741 | -0.4 |
| [a12]+ | 1378.6023 | 1378.6025 | 0.1 |
| [a13]+ | 1515.6612 | 1515.6624 | 0.8 |
| [a13]2+ | 758.3343 | 758.3347 | 0.6 |
| [a14]2+ | 826.8637 | 826.8637 | 0.0 |
| [a20]2+ | 1208.0851 | 1208.0838 | -1.1 |
| [a24]2+ | 1415.1727 | 1415.1732 | 0.4 |
| [a32]3+ | 1190.9182 | 1190.9171 | -1.0 |
| [c7]+ | 888.3959 | 888.3954 | -0.6 |
| [c25]2+ | 1466.1941 | 1466.1946 | 0.3 |
| [c25]3+ | 977.7985 | 977.7974 | -1.1 |
| [y7]+ | 614.3872 | 614.3878 | 0.9 |
| [y8]+ | 745.4277 | 745.4288 | 1.4 |
| [y10]+ | 915.5332 | 915.5326 | -0.7 |
| [y11]+ | 1028.6173 | 1028.6164 | -0.9 |
| [b14+Co(II)]2+ | 869.3199 | 869.3193 | -0.7 |
| [b24+Co(II)]3+ | 972.0884 | 972.0872 | -1.2 |
| [b31+Co(II)]3+ | 1181.1997 | 1181.1989 | -0.7 |
| [b32+Co(II)]3+ | 1218.8944 | 1218.8951 | 0.6 |
| [b34+Co(II)]3+ | 1275.5962 | 1275.5964 | 0.1 |
| [b35+Co(II)]3+ | 1319.2764 | 1319.2755 | -0.7 |
| [b36+Co(II)]3+ | 1352.2992 | 1352.2999 | 0.5 |
| [b38+Co(II)]3+ | 1390.3135 | 1390.3129 | -0.5 |
| [b39+Co(II)]3+ | 1423.3363 | 1423.3388 | 1.8 |
| [b40+Co(II)]3+ | 1456.3591 | 1456.3609 | 1.2 |
| [b34+Co(II)]4+ | 956.9490 | 956.9495 | 0.5 |
| [b35+Co(II)]4+ | 989.7091 | 989.7086 | -0.6 |
| [b37+Co(II)]4+ | 1028.7316 | 1028.7316 | 0.0 |
| [b39+Co(II)]4+ | 1067.7541 | 1067.7533 | -0.7 |
| [a13+H+Co(II)]2+ | 787.2967 | 787.2968 | 0.2 |
| [a14+Co(II)]2+ | 855.3225 | 855.3214 | -1.3 |
| [a24+Co(II)]3+ | 962.7567 | 962.7567 | 0.0 |
| [a26+H+Co(II)]3+ | 1011.1103 | 1011.1084 | -1.9 |
| [a25+H+Co(II)]4+ | 982.0997 | 982.0997 | 0.1 |
| [a39+Co(II)]4+ | 1060.7553 | 1060.7554 | 0.1 |
| [a40+H+Co(II)]4+ | 1085.7743 | 1085.7744 | 0.1 |
| [a42+H+Co(II)]4+ | 1131.8046 | 1131.8047 | 0.2 |
| [c25+Co(II)]3+ | 996.7710 | 996.7708 | -0.3 |
| Average |  |  | -0.06 |
| St Dev |  |  | 0.9 |

**Supporting Information S7**: Fragment assignment for native top-down MS of the peptide-metal complexes investigated in this study. All listed *m/z* values are monoisotopic.
